# Buried with their Buckles On: Clothed Burial at the Augustinian Friary, Cambridge

**DOI:** 10.1080/00766097.2022.2065066

**Published:** 2022-06-13

**Authors:** Craig Cessford, Andrew Hall, Bram Mulder, Benjamin Neil, Ian Riddler, Justin Wiles, with CONTRIBUTIONS BY, Esther Cameron, Quita Mould

## Abstract

GIRDLE BUCKLES ASSOCIATED with several groups of burials at the later-medieval Augustinian friary in Cambridge indicate that clothed burial was common, with a much higher prevalence than usual for the period. The clothed burial was highly regularised, in terms of both the garments worn and the types of buckles used, and apparently limited to members of the Augustinian Order. The buckles were primarily supplied by the friary, rather than representing individual choices, and there is evidence that the girdles that individuals were buried with were those they used in life. These buckles functioned on several levels, as symbols of both corporate and personal identity. Discoveries at other English Augustinian friaries demonstrate specific typological parallels, indicating broader connections, networks and identities. Although buckles are common late-medieval items, those from the Cambridge Augustinian friary present a unique opportunity to contextualise their use by one segment of society.

## Résumé

**Inhumés avec leur ceinture : inhumations habillées au monastère des Frères de l’ordre de Saint-Augustin, à Cambridge** *par* Craig Cessford, Andrew Hall, Bram Mulder, Benjamin Neil, Ian Riddler *et* Justin Wiles, *avec les contributions d’*Esther Cameron *et de* Quita Mould

Les boucles de ceinture associées à plusieurs groupes de sépultures de la fin du Moyen-Âge, au monastère des Frères de Saint-Augustin à Cambridge, indiquent que l’inhumation des corps habillés était pratiquée couramment, bien plus que ce n’était usuel à cette période. L’inhumation des défunts habillés était hautement codifiée, aussi bien en termes des vêtements portés et des types de ceinture, et se limitait apparemment aux membres de l’ordre des Frères de Saint-Augustin. Les boucles étaient fournies principalement par le monastère, plutôt qu’un choix individuel, et certains éléments suggèrent que les ceintures portées par les défunts étaient celles qu’ils portaient dans la vie. Ces boucles étaient symboliques à plusieurs niveaux, à la fois d’une identité corporatiste et individuelle. Les découvertes dans d’autres monastères anglais de l’ordre des Frères de Saint-Augustin présentent des parallèles typologiques spécifiques, indiquant plus largement des liens, des réseaux et des identités. Bien que les boucles soient couramment retrouvées à la fin du Moyen-Âge, celles du monastère des Frères de Saint-Augustin à Cambridge nous donnent une unique opportunité de contextualiser leur utilisation par un segment de la société.

## Zusammenfassung

**Gräber mit Gürtelgarnituren: Bekleidete Bestattung im Augustinerkloster, Cambridge** *von* Craig Cessford, Andrew Hall, Bram Mulder, Benjamin Neil, Ian Riddler *und* Justin Wiles, *mit Beiträgen von* Esther Cameron *und* Quita Mould

Gürtelgarnituren aus mehreren Gräbergruppen des spätmittelalterlichen Augustinerklosters in Cambridge deuten darauf hin, dass die Beisetzung in Kleidern dort üblich war und einen für die damalige Zeit ungewöhnlich hohen Verbreitungsgrad hatte. Die Beerdigung in Kleidern war sowohl in Bezug auf die getragenen Kleidungsstücke als auch auf die Art der verwendeten Schnallen stark reglementiert und offenbar auf Mitglieder des Augustinerordens beschränkt. Die Schnallen wurden vorwiegend vom Kloster beschafft und stellten keine individuelle Wahl dar. Es gibt jedoch Hinweise darauf, dass es sich bei den Gürteln, mit denen die Personen bestattet wurden, um diejenigen handelte, die sie zu Lebzeiten getragen hatten. Die Schnallen fungierten auf mehreren Ebenen als Symbole sowohl der korporativen als auch der persönlichen Identität. Funde in anderen englischen Augustinerklöstern zeigen spezifische typologische Parallelen, die auf umfassendere Verbindungen, Netzwerke und Identitäten hinweisen. Gürtelschnallen waren im Spätmittelalter zwar weit verbreitet, die aus dem Augustinerkloster in Cambridge stammenden Schnallen bieten jedoch eine einzigartige Gelegenheit, ihre Verwendung seitens einesbestimmten Gesellschaftssegments zu kontextualisieren.

## Riassunto

**Sepolti con le proprie fibbie: sepolture vestite nel convento agostiniano di Cambridge** *di* Craig Cessford, Andrew Hall, Bram Mulder, Benjamin Neil, Ian Riddler *e* Justin Wiles, *con contributi di* Esther Cameron *e di* Quita Mould

Le fibbie per cintura associate a diversi gruppi di sepolture presso il convento agostiniano tardomedievale di Cambridge indicano che la sepoltura vestita era una pratica usuale, con una prevalenza maggiore del solito in quel periodo. La sepoltura vestita seguiva regole molto precise sia per quanto riguarda gli indumenti indossati, sia per le fibbie impiegate e, a quanto pare, era limitata ai membri dell’ordine agostiniano. Le fibbie venivano principalmente fornite dal convento anziché rappresentare scelte individuali e risulta che le cinture con cui erano sepolti gli individui erano quelle che usavano da vivi. Queste fibbie avevano molteplici valenze come simboli sia di identità personale che collegiale. I ritrovamenti presso altri conventi agostiniani inglesi dimostrano specifici paralleli tipologici, il che indica l’esistenza di legami, reti e identità più estesi. Benché le fibbie siano oggetti comuni nel Basso Medioevo, quelle del convento agostiniano di Cambridge offrono la straordinaria possibilità di contestualizzarne l’uso da parte di un settore della società.

Buckles were used in Britain from the Romano-British period, but from the 12th–13th centuries onwards the quantity in use and range of forms increased markedly. The wearing of belts began around the age of seven and most adults of both sexes would have worn belts fastened with mass produced metal buckles, with evidence of up to 144 buckle frames being cast together.^9^ Although there have been numerous studies of later-medieval (11th–15th century) buckles, these have generally focused on typological characterisation and dating, and less attention has been paid to other aspects of these items.^10^ There are two principal reasons for this typological focus. The main factor is that most buckles are recovered from depositional contexts that are difficult to link to their original contexts of usage. A prime example of this is the London waterfront sites, which form the basis of the standard typological study.^11^ Such waterfront assemblages comprise commingled waste material from multiple sources, covering an unknown range of places in the city, possibly including public rubbish tips.^12^ While buckles from other urban contexts sometimes derive from more informative contexts, these are often residual and rarely considered in detail.^13^ Examples of even more poorly contextualised assemblages include the unstratified finds or metal-detected items from rural sites recorded by the Portable Antiquities Scheme.^14^ This means that it is generally impossible to associate most buckles with particular individuals, or even groups.

Compounding this issue, buckles are multi-functional objects used to fasten either two straps or two ends of a single strap, consisting of a frame that the strap passes through and a pin that goes through a hole in the strap. Later-medieval buckles were used on numerous different items of clothing, as well as shoes, horse harnesses, armour, spurs, dog leads, hawks’ jesses, satchels etc. While specific functions can be suggested for a few, generally relatively rare, forms, most buckles cannot be linked to a specific function and many may have been multi-functional. Additionally, it is not always possible to distinguish buckles from brooches, and one buckle from the site appears to be a repurposed brooch.

More recently, large assemblages of mainly poorly contextualised buckles and other dress accessories from both rural and urban sites have been examined by scholars in an attempt to understand them better.^15^ While useful, such studies are largely limited to broad typologically driven comparisons. Broad distinctions such as religious versus domestic are sometimes possible at the level of sites.^16^ However, these are questionable as secular individuals were present on religious sites, and items used by religious individuals were surely disposed of at non-religious sites. An alternative approach which may overcome these issues of identification is to consider buckles found in contexts that allow them to be more directly associated with specific individuals or groups. Some case studies have successfully associated late-medieval assemblages with particular households or individuals, but these are relatively uncommon and dress accessories are rare and the function of some objects is often hard to determine.^17^

Broadly speaking, the contribution of funerary evidence is also limited, hampered especially by a lack of clothed burials; typically, only 2–3% of later-medieval individuals are found interred with dress accessories.^18^ There are, however, some circumstances in which clothed burial was more common, such as the Cambridge Augustinian friary, which forms the focus of this study. The buckles indicate that clothed burial was relatively common and highly regularised at the friary, and their use seemingly restricted to members of the Augustinian Order. A limited range of buckle forms are represented; some particularly common forms differ from those in use by the wider community in Cambridge and the county, suggesting that they were supplied by the order rather than through individual acquisition. The buckles were made from a range of materials, including relatively uncommon bone and ivory examples. The evidence recovered from Cambridge also appears to be reflective of a national trend, with clothed burials and comparable buckle types found at Augustinian friaries elsewhere in England. It will be argued that these buckles functioned as symbols of both corporate and individual identity on several levels.

## AUGUSTINIAN FRIARS’ DRESS

Clothing was significant to members of late-medieval religious orders, acting to distinguish them from the laity and more specifically allowing them to be identified as members of a specific order and stressing its collective unity.^19^ For Augustinian friars this proved particularly controversial, as it was claimed their dress was based upon that of St Augustine himself.^20^ The prescribed dress of the Order was laid down when it was created in 1256, when Pope Alexander IV issued the papal bull *Licet ecclesiae catholicae*. The Regensburg or Ratisbon Constitutions, the fundamental body of Augustinian rules, drawn up in the 1290s, paid considerable attention to clothing, with chapter 24 on ‘The number and quality of the brothers clothing’ [*De numero et qualitate vestium Fratrum*].^21^ This states that the girdle was to be worn visibly, made of black leather and should not be more than two or less than one and a half fingers wide. This concern continued into the fourteenth century, with Jordan of Quedlinburg/Saxony in his ‘Life of the Brethren’ (*Liber vitasfratum)* of 1357 devoting a chapter to the habit worn by the Order (*De habitu ordinis:* book I, chapter 15).^22^ Augustinian writers considered the black cowl, a large loose hood, and leather girdle to be the principal visual signifiers of their Order, and these dominate artistic depictions.

The adoption of the girdle initially related to its eremitic associations, as the Augustinians developed from various groups of hermits. It retrospectively acquired various symbolic explanations that are mentioned in the writings of various members of the Order such as Jordan of Quedlinburg. Black indicated awareness one was a sinner or contempt for the adornments and beauty of the world, while the wearing of part of a dead animal in the leather girdle signified the putting to death of animal instincts, especially in those parts that contain the source of lust.^23^

Although textiles do not survive well (see below), the cowls that were the other principal visual signifier of Augustinian friars may also be archaeologically identifiable. Seven individuals from the friary had probable infections of their scalp. These are predominantly individuals with associated buckles, or where truncation means that buckles may have been present. These infections possibly relate to tonsuring practices and wearing cowls may have perpetuated/aggravated such infections.^24^

Theoretically, mendicant clothing, including girdles, was communally owned and handed out to members according to their needs. There is no evidence that friaries produced their own girdles, and it is likely that most were purchased locally, although it is possible that some clothing was acquired as charitable gifts from benefactors. Articles of clothing were redistributed to other brethren after death, but importantly corpses were washed and clothed before burial. Selection of dress therefore lay not with the deceased but with those undertaking these corporeal acts, and suggests that girdles and their associated buckles were carefully chosen for internment. It is notable that the same level of care was taken over clothing the body even in cases of violent death or when individuals had died during outbreaks of plague.^25^

Although like all mendicants the English Augustinian friars were committed to poverty, they never embraced contemporary concepts of extreme poverty, such as the doctrines of Apostolic Poverty and Absolute Poverty of Christ. Instead, they generally advocated moderation and avoidance of the extremes of asceticism or poverty and thus had no issues with the retention of communal property, or of some level of individual property.^26^ Robert Barnes, the early 16th-century reformer and prior of the Cambridge Augustinian friary, argued against absolute poverty stating that possessions are not inherently abhorrent, just the extent to which the clergy accrues them.^27^ Although most of the evidence for clothing comes from continental sources, when describing St Augustine’s clothing, Osbern Bokenham (c 1393–1464), an English Augustinian friar and poet, wrote, ‘His clothing, his hose, his shoes and his other ornaments were neither too shining nor too abject, but of moderate and competent habit’.^28^ This reflects the exhortation in the Rule of St Augustine, ‘Do not allow your clothing to attract attention; seek to please not by the clothes you wear, but by the life you live’.^29^

## THE AUGUSTINIAN FRIARY, CAMBRIDGE

**Fig 1:**
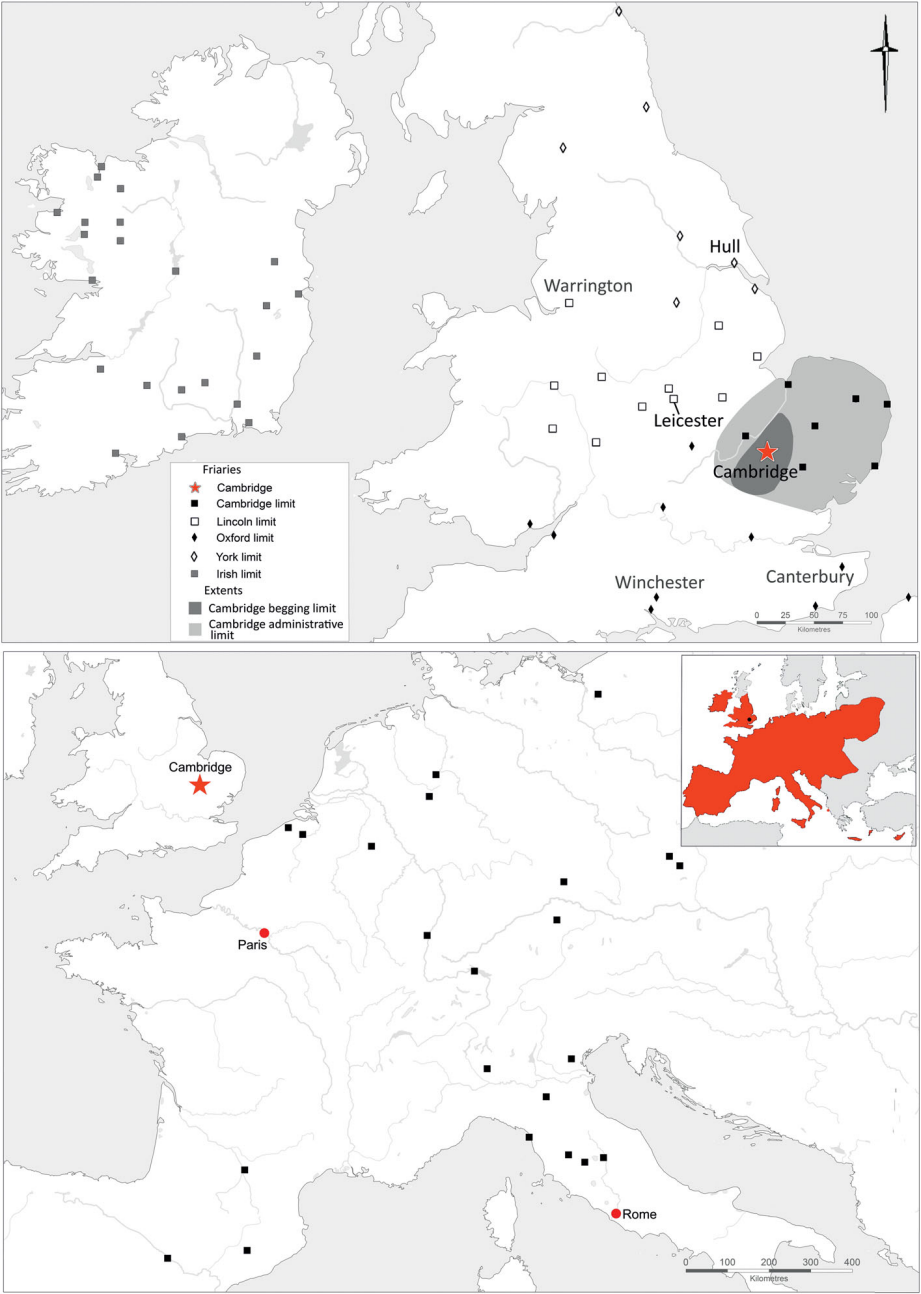
The Cambridge Augustinian friary in a broader context. Location map showing Augustinian friaries in the English and Irish national province, plus approximate extent of the begging/alms limit and the administrative limit of the Cambridge Augustinian friary (upper). Origins of continental friars documented as spending time in Cambridge (black squares), and the principal *studium generale* of the order in Paris, and the headquarters of the order in Rome plus inset showing extent of the Augustinians up to 1538 in red (lower). *Plotted by David Redhouse for the After the Plague* *project and modified by Andrew Hall.*

**Fig 2:**
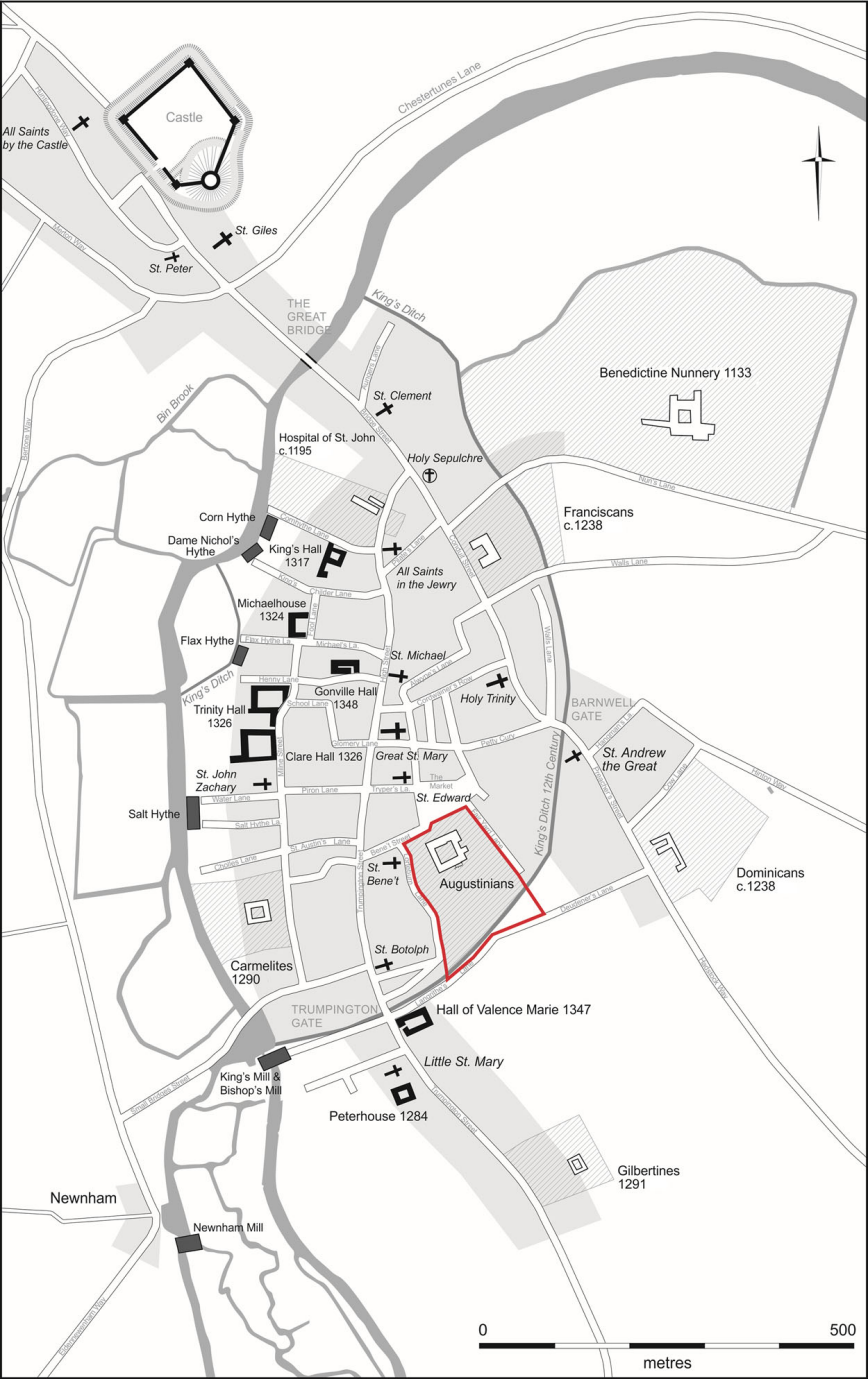
Plan of Cambridge c 1350 with location of Augustinian friary. *By Vicki Herring for the After the Plague project*.

**Fig 3:**
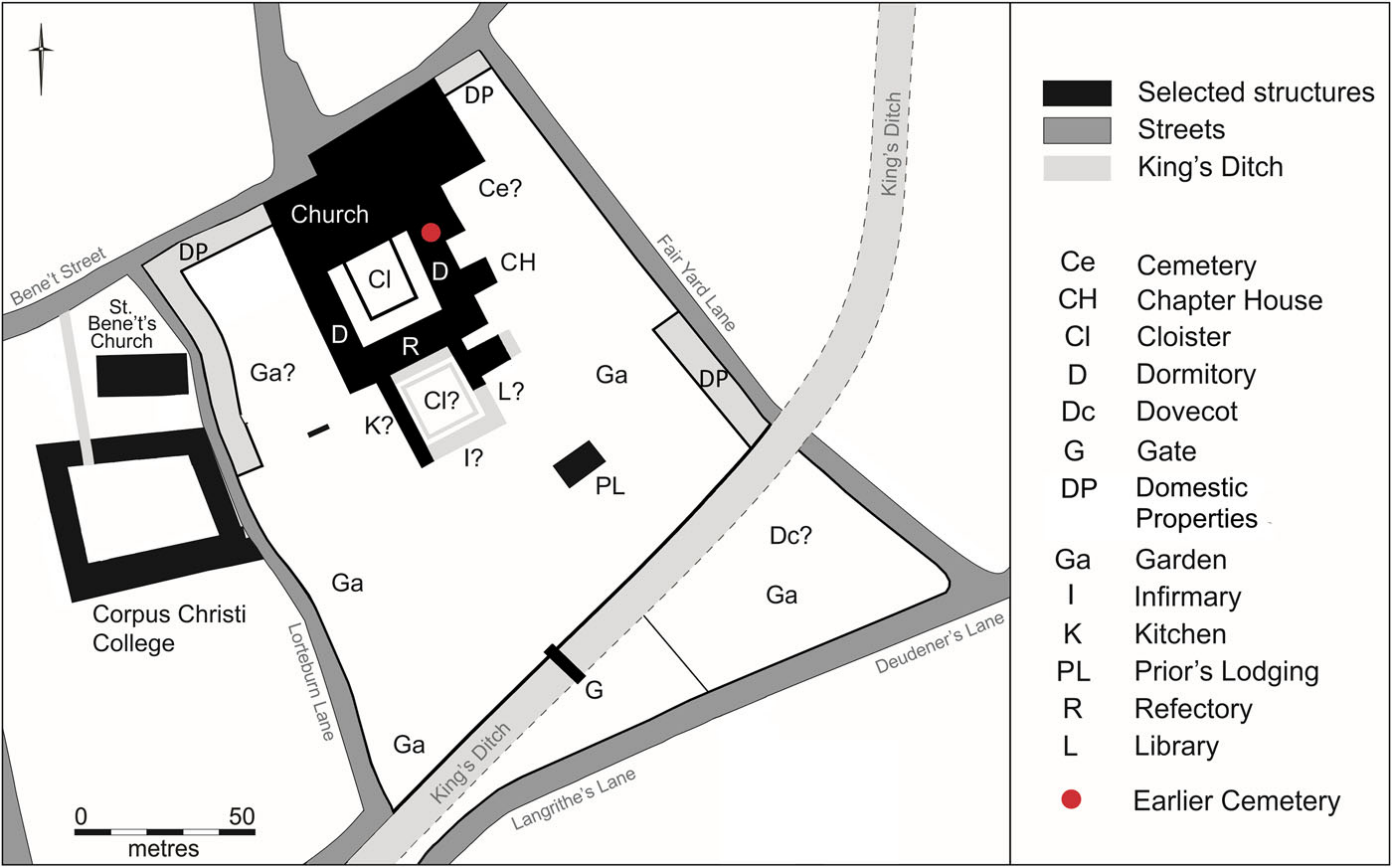
Reconstructed plan of the later phase of the Cambridge Augustinian friary, also showing location of the earlier cemetery. *By Laura Hogg and Andrew Hall, © Cambridge Archaeological Unit*.

**Fig 4:**
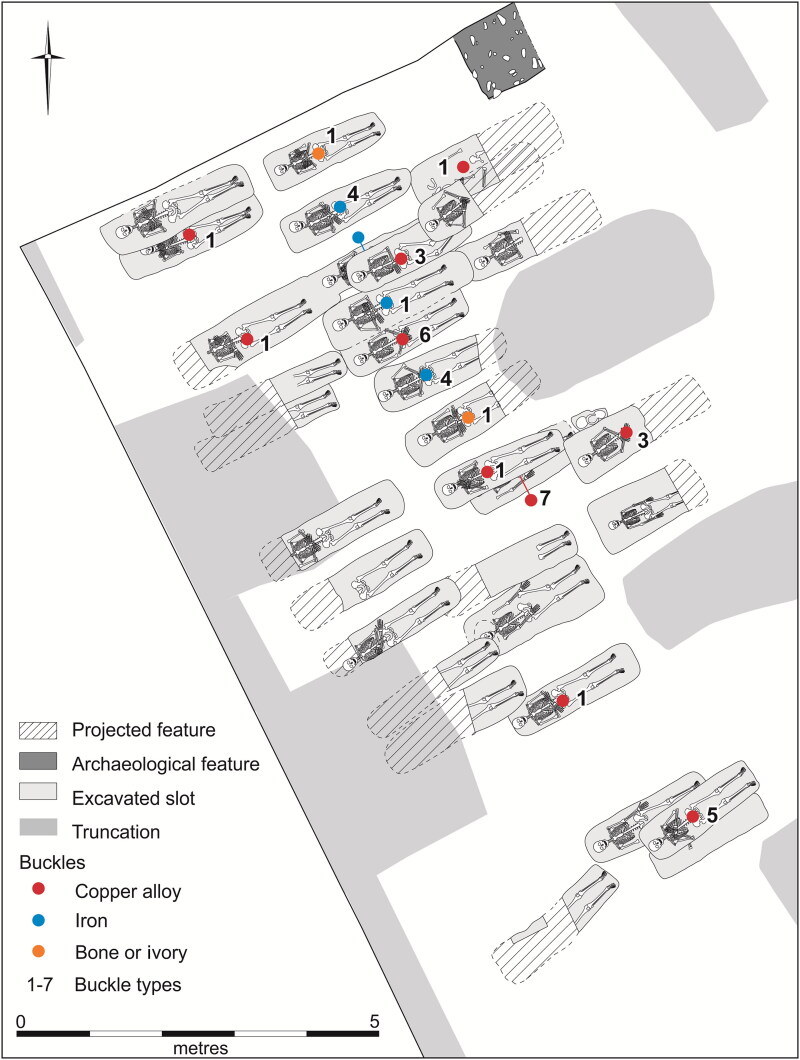
Plan of Cambridge Augustinian friary cemetery, showing buckle types and materials. *By Andrew Hall, © Cambridge Archaeological Unit*.

**Fig 5:**
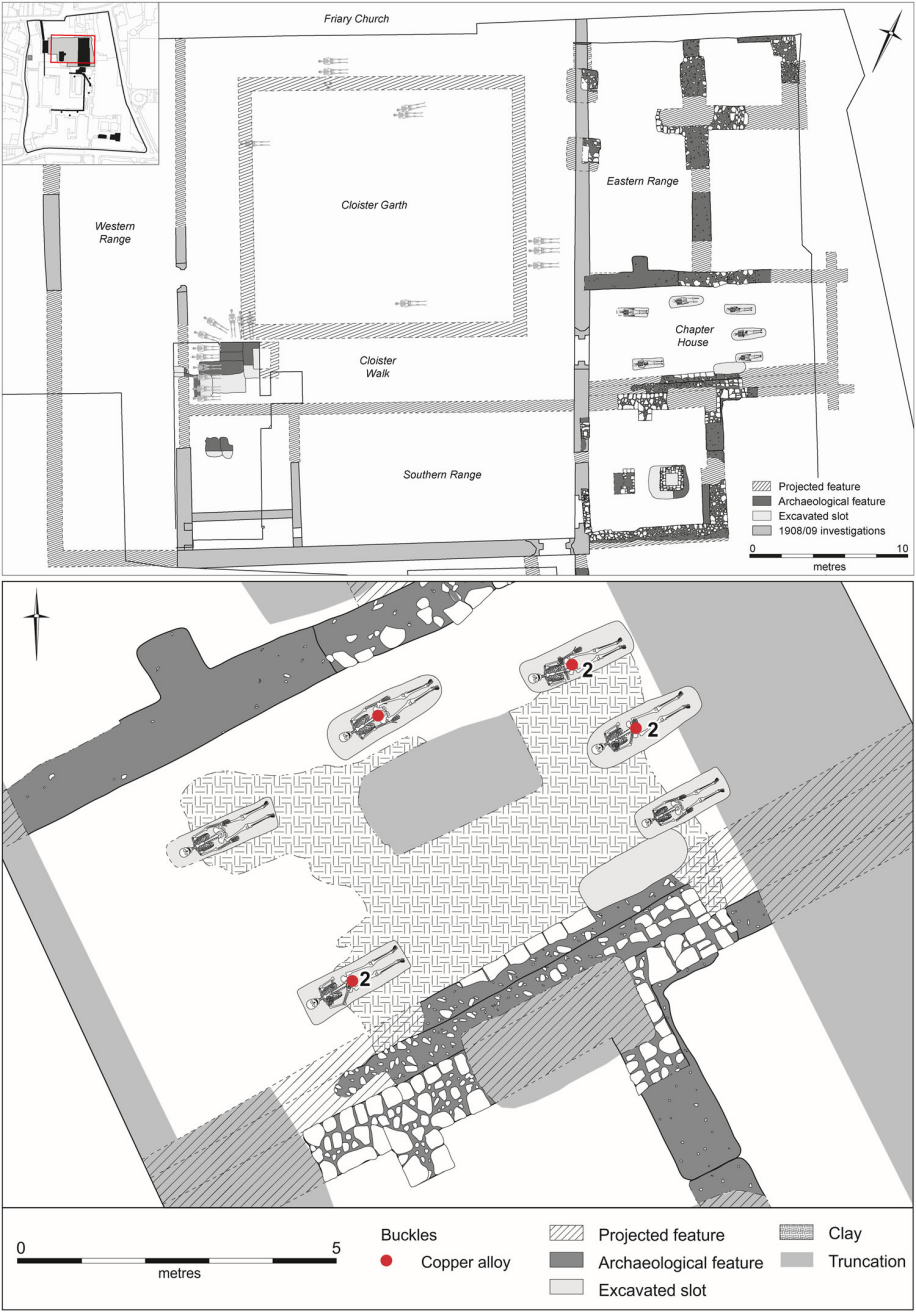
Plan of later Cambridge Augustinian friary cloister combining information from 1908–9 and 2016–17 (upper), and plan of chapter house, showing buckle types and materials (lower). *By Andrew Hall and Laura Hogg, © Cambridge Archaeological Unit*.

The Augustinian friary in Cambridge (Figs 1–3), was in many respects a typical example of the mendicant houses established across the urban centres of the Western Church in the 13th century.^30^ The Hermit Friars of St Augustine (*Ordo Eremitorum Sancti Augustini*), commonly referred to as the Augustinian or Austin friars, were created by the Great Union of 1256, although some groups that formed this were present in Britain as early as 1249. The Cambridge friary was established 1279/80–89 and appears to have been, ‘firmly settled by 1289’, suggesting a date of c 1279/80–85.^31^ Cambridge was a medium-sized market town and inland port with a population of c 3000, which had developed into a notable centre of learning from the 1220s. The Augustinians joined houses of other mendicant orders, such as the Dominicans, Franciscans, Carmelites, the Friars of St Mary and the Friars of the Sack. The Augustinian friary grew rapidly and thrived — holding meetings of the heads of the English Augustinian friaries from 1316 onwards, becoming a *studium generale* or international study house in 1318 and having 70 friars present in 1328 — and continued until the Dissolution in 1538. The friary probably acquired burial rights for members of the Order in 1290, when it agreed to compensate the parish of St Edward and its patron Barnwell Priory.^32^ In 1302 the friary was granted rights of burial, preaching and hearing confessions for those who were not members of the community.^33^ Three groups of burials associated with the friary have been excavated. In 2016–17, 32 individuals from an early cemetery south of the church (Fig 4), and six individuals from a later chapter house were excavated (Fig 5).^34^ Earlier excavations in 1908–9 revealed 30+ skeletons, these probably relate to the cloister walk and garth and are contemporary with the chapter house (Fig 5).^35^

The cemetery must date to after the friary acquired burial rights in 1290 and was still in use in 1349, as one skeleton tested positive for *Yersinia pestis* and must have died during the Black Death at the earliest.^36^ Inspection of single nucleotide polymorphisms classified it as potentially identical to other Black Death genomes, as these changed rapidly. Evidence for the cemetery continuing after this burial indicates that the earliest possible end date is c 1360. The cloisters were built over the cemetery, with their footings truncating several burials including the one that tested positive for *Yersinia pestis*. Blocks from the cloister arcade, which were reused in a later nearby cellar, and other elements stylistically date to after c 1330 and although their latest possible date is much less certain they would normally be assumed to be no later than c 1370. Modelling and Bayesian analysis of the radiocarbon determinations is not in agreement with this, with only a 2.5% likelihood that the cemetery went out of use by 1390. Instead, these suggest that the cemetery probably went out of use c 1400–40 (89.3% likelihood). Given the discrepancies in the evidence, the cemetery is dated to c 1290–1360/1440 and the chapter house and cloister walk burials to c 1360/1440–1538. Individual burials can frequently be dated more precisely using a range of types of evidence (Tab 1).^37^

**Table 1:** Burials from the 2016–17 excavations at the Cambridge Augustinian friary.

Feature	Location	Age at death	Date of death	Buckle	Buckle position	Buckle orientation	Interpretation and comments
106	Cemetery	Infant: 0–4	1310–49	No	–	–	Female child of benefactor
140	Cemetery	Young mid adult: 26–35	1302–49	No	–	–	Benefactor
146	Chapter house	Mature adult: 46+	1500–38	No	–	–	Benefactor, female
190	Chapter house	Juvenile: 5–12	1425/75–1538	No	–	–	Child of benefactor. Died of plague
191	Chapter house	Mature adult: 46+	1425/75–1538	Type 2, copper alloy	Pelvis, upper wing of left ilium	Right	Adult friar. Possible scalp infection
195	Cemetery	Adult: 18+	1290–1349	Unknown	–	–	Uncertain if Augustinian or laity
196	Cemetery	Adult: 18+	1302–49	Unknown	–	–	Uncertain if Augustinian or laity
198	Cemetery	Mature adult: 46+	1302–49	No	–	–	Benefactor
199	Cemetery	Adult: 18+	1290–1349	Unknown	–	–	Uncertain if Augustinian or laity
215	Cemetery	Mature adult: 46+	1290–1349	Unknown	–	–	Uncertain if Augustinian or laity
216	Cemetery	Old middle adult: 36–45	1300–49	Type 1, copper alloy	L5 vertebra	Right	Young adult friar, possibly studying for advanced degree
217	Cemetery	Adult: 18+	1290–1349	Unknown	–	–	Uncertain if Augustinian or laity
230	Chapter house	Juvenile: 5–12	1475–1538	Type 2, copper alloy	Pelvis, upper wing of left ilium	Right	Novice. Died of plague
232	Cemetery	Younger old middle adult: 36–45	1320–49	No	–	–	Benefactor
237	Cemetery	Younger old middle adult: 36–45	1290–1360/1420	Unknown	–	–	Uncertain if Augustinian or laity
260	Chapter house	Young adult: 18–25	1360/1420–1425/75	Type unknown, copper alloy	L5 vertebrae	Unknown	Young friar, possibly studying for first degree. Possible scalp infection
265	Cemetery	Younger old middle adult: 36–45	1320–49	Type 1, copper alloy	Pelvis, over pubic arch	Right	Adult friar
302	Cemetery	Younger old middle adult: 36–45	1300–1360/1420	Type 5, copper alloy	Sacrum, central on S1	Right	Senior friar
309	Cemetery	Young mid adult: 26–35	1290–1360	Unknown	–	–	Uncertain if Augustinian or laity. Possible scalp infection
310	Chapter house	Young adult: 18–25	1425/75–1538	Type 2, copper alloy	To right of L3 vertebra	Right	Young friar, possibly studying for first degree. Died of plague
311	Cemetery	Mature adult: 46+,	1290–1349	Type 4, iron	Pelvis, upper wing of left ilium	Right	Senior friar
312	Cemetery	Adolescent: 13–18	1302–49	No			Child of benefactor
314	Cemetery	Younger young adult: 18–25	1302–49	Type 1, ivory	Sacrum, right side of S2	Left	Young friar, possibly studying for first degree
315	Cemetery	Young adult: 18–25	1302–49	Unknown	–	–	Uncertain if Augustinian or laity
328	Cemetery	Adolescent: 13–18	1349–1360/1420	Unknown	–	–	Uncertain if Augustinian or laity. Possible scalp infection
331	Cemetery	Old middle adult: 36–45	1320–49	Type 3, copper alloy	To right of L5 vertebra	Right	Adult friar
332	Cemetery	Young adult: 18–25	1349–1360/1420	Type 3, copper alloy	L4 vertebra	Upwards	Young adult friar, possibly studying for advanced degree. Probable fatal accident
333	Cemetery	Young middle adult: 26–35	1290–1349	Type 3, iron	Sacrum, S1	Right	Adult friar
334	Cemetery	Old middle adult: 36–45	1320–49	Type 1, copper alloy	L5 vertebra	Right	Senior friar
336	Cemetery	Mature adult: 45+	1300–49	Type 1, copper alloy	Left side of L4 vertebra	Right	Senior friar
343	Cemetery	Adolescent: 13–18	1309–49	Type unknown, iron	Uncertain	Right	Young friar, possibly studying for first degree. Possible scalp infection
344	Cemetery	Old Middle Adult: 36–45	1320–49	Type 7, copper alloy	L5 vertebra	Right	Adult friar
346	Cemetery	Juvenile: 5–12	1290–1349	Unknown	–	–	Uncertain if Augustinian or laity
347	Cemetery	Adolescent: 13–18	1300–49	Type 1, bone	Sacrum, central on S1	Right	Novice or young local friar
348	Cemetery	Young middle adult: 26–35	1349–1360/1420	Type 1, iron	Sacrum, central on S2	Right	Adult friar
352	Cemetery	Young mid adult: 26–35	1349–1360/1420	Type 1, copper alloy	Unknown	Unknown	Heavily disturbed skeleton. Young adult friar, possibly studying for advanced degree. Possible scalp infection
355	Cemetery	Young mid adult: 26–35	1349	Probably none (slightly ambiguous truncation)	–	–	Benefactor, died of plague. Possible scalp infection
367	Cemetery	Mature adult: 45+	1300–49	Type 6, copper alloy	Between sacrum and pelvic pubic arch	Right	Adult friar

Sex is male unless otherwise indicated. Interpretation of individuals based on age at death, sex of skeletons and presence or absence of girdle buckles.^92^

**Fig 6:**
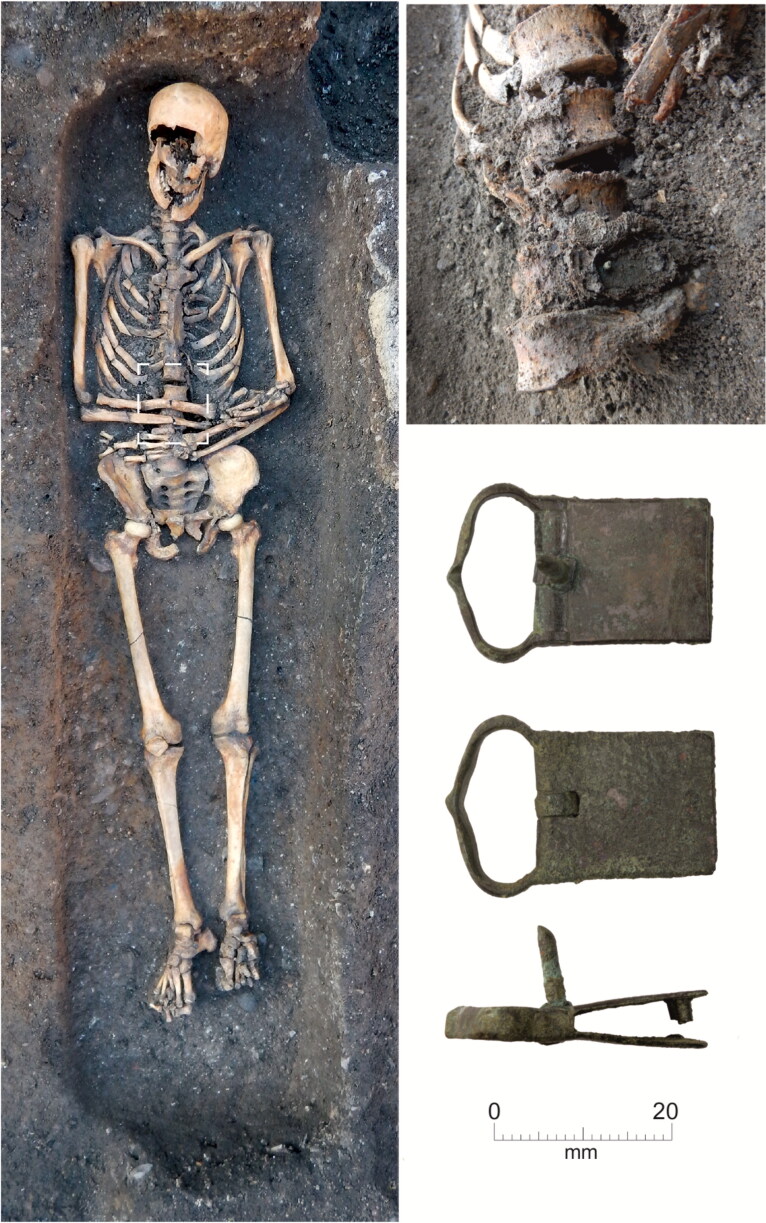
Cambridge Augustinian friary, burial F.336 plus in situ detail of buckle. Type 1 copper alloy buckle pointing right. *Photographs by Craig Cessford and Dave Webb, © Cambridge Archaeological Unit*.

**Fig 7:**
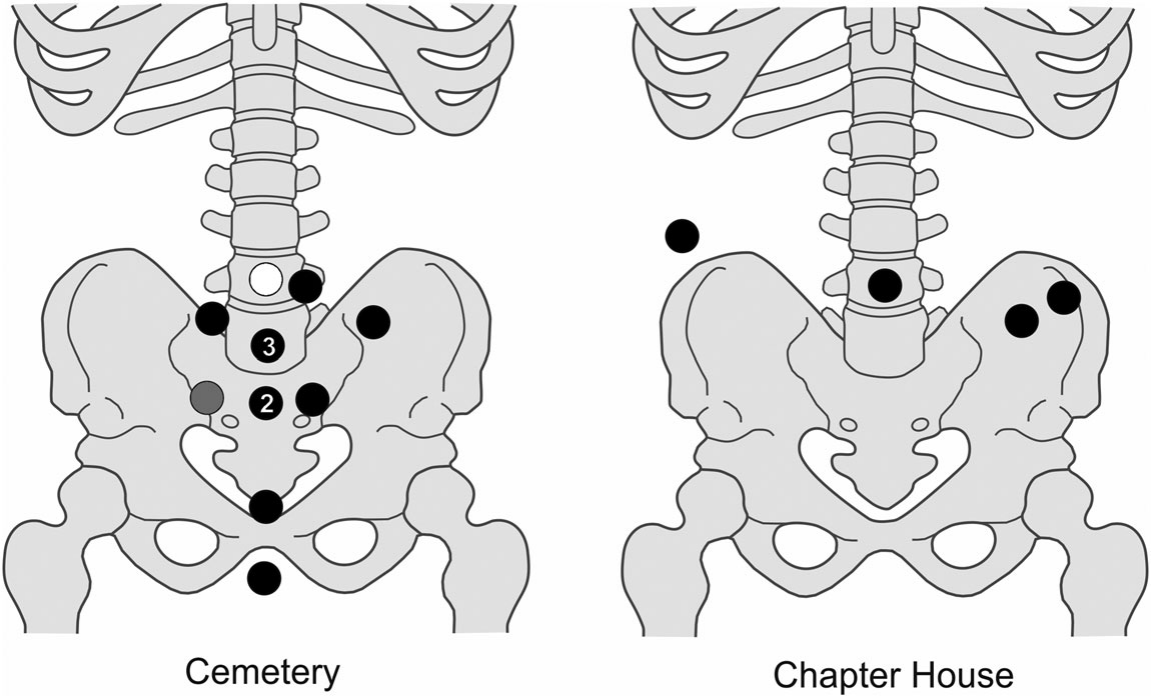
Cambridge Augustinian friary, locations of buckles on associated skeletons at cemetery and chapter house. Black: buckle pointing right; grey: buckle pointing left; white: buckle pointing vertically. *By Andrew Hall © Cambridge Archaeological Unit*.

The burials, aligned WSW/ENE, were extended supine inhumations in simple earth-cut graves, with the head to the west. All three excavated groups contain both clothed burials with girdle buckles and others without them, who were presumably buried unclothed in shrouds. Although dress accessories are infrequent in burials of this period, a wide range of types has been found. These include pairs of large annular buckles located between the thigh and hip and interpreted as ‘breche’ or hose buckles, lace tags, shoe buckles, brooches and rings.^38^ All the clothed burials at the Cambridge Augustinian friary had only a single buckle at the waist (Figs 6–7), indicating that this was part of a girdle or belt. The term girdle is preferred to belt, to distinguish these as probable items of religious or liturgical attire. There is also no evidence that any of the girdles were particularly ornate with additional mounts etc. Although shoe buckles are known from other late-medieval burials, none were found at the friary. This accords with textual evidence that friars’ shoes were to be, ‘black and fastened by a thong at the ankle’.^39^ No direct evidence for shoes survived in the graves, this is however unsurprising as the evidence of the girdles indicates that leather only survived where it was in contact with items of metalwork. The positions of the feet of the skeletons with associated girdle buckles all appear compatible with them wearing shoes. Overall, the nature of clothed burial at the friary was highly regularised and uniform.

Most of the individuals were adolescent or adult males, indicating that a significant proportion, but not all of those buried, were members of the Augustinian Order. None of the individuals who cannot have been members of the Order, either due to their sex (ie females) or because they were too young (under 14 prior to the Black Death, and ten or 11 afterwards) had buckles associated with them.^40^ Taken together, the uniformity of the dress accessories plus the sex and age pattern associated with them indicates that the clothed burials were Augustinians. The members of the Order can be broadly placed into several age-based groups. Of particular significance to the friars was the fact that St Augustine divided life into six stages, to which Isidore of Seville added specific ages.^41^ These were *infantia* (infancy, 0–7 years), *pueritia* (childhood, 7–14 years), *adolescentia* (adolescence 15–28 years), *iuventus* (youth or manhood 29–49 years), *aetas senioris* or *gravitas* (elder 50–69 years), and *senectus* (old age or decrepitude, 70+). Prior to the Black Death those aged c 14–20 would be undertaking their novitiate and their initial studies, which comprised a year studying grammar or logic and three years studying philosophy. Those aged c 20–24 would either be relatively junior members of the Cambridge friary or undertaking an initial degree at the *studium generale*. Twenty-four was a significant age, as in 1287 the international general chapter of the Order ruled that no friar was to be promoted to the priesthood under this age and this was not reduced until 1507. Friars aged c 24–30 might either be members of the Cambridge friary or visitors studying for a more advanced degree. Friars aged over c 50 might be regarded as senior members of the friary, even if they did not occupy a specific role. Although several burials that lack buckles are of the appropriate sex and age to have been Augustinians, it is likely that many of the lay benefactors buried at the friary were adult males so these may well be laypeople. They are most likely to be benefactors, or for younger individuals the offspring of benefactors who died in childhood. If clothed burial was associated with Augustinians, it is possible that some individuals with buckles took orders on their deathbed, becoming religious after a previously lay life.^42^

## THE BUCKLES

Of the 32 burials from the cemetery of c 1290–1360/1440 16 (50%) had buckles (Tab 1).^43^ Several skeletons had been truncated in a way that made it impossible to know if they originally had an associated buckle or not. When these are removed from consideration, 16 of the remaining 22 skeletons had a buckle (73%). Only six individuals were buried in the subsequent chapter house of c 1360/1440–1538. Four of these had buckles, indicating a similar prevalence rate of two thirds. The burials recovered in 1908–9 appear to relate to the cloister walk, which was in existence for the same period as the chapter house. It is impossible to be certain, but it appears that parts of at least 47 skeletons were recovered along with a minimum of eight buckles (23.5%, although this may underrepresent the original prevalence).^44^ Evidence for the leather girdle straps and other clothing from the Cambridge friary is limited.^45^ Apart from the girdle buckles few other dress accessories were recovered and none are conclusively contemporary with the friary.

The buckles include a range of forms and materials. Although most buckles were made of copper alloy (22), there were also some of iron (4), plus single examples of both elephant ivory and animal bone. Copper alloy and iron were relatively common materials for manufacturing buckles in late-medieval England, whereas ivory and bone were much rarer. Examples from London include 178 of copper alloy, 184 of lead/tin and 115 of iron, with none of bone or ivory.^46^ It has been argued that lead/tin buckles are rare outside London, although mounts, shoe buckles and badges of this material appear to be common in 14th–16th-century urban groups.^47^ They are, however, rare in the town of Cambridge and not present at the friary. The four iron buckles occur in adjacent burials in a single row of the cemetery (see Fig 4). As individuals were buried consecutively by row, it appears that iron buckles only accompanied burials for a short time. The bone and ivory buckles accompanied burials in the same row, separated by the four individuals with iron buckles plus one with a copper alloy buckle. This suggests a relatively brief period when materials other than copper alloy were in use.

**Fig 8:**
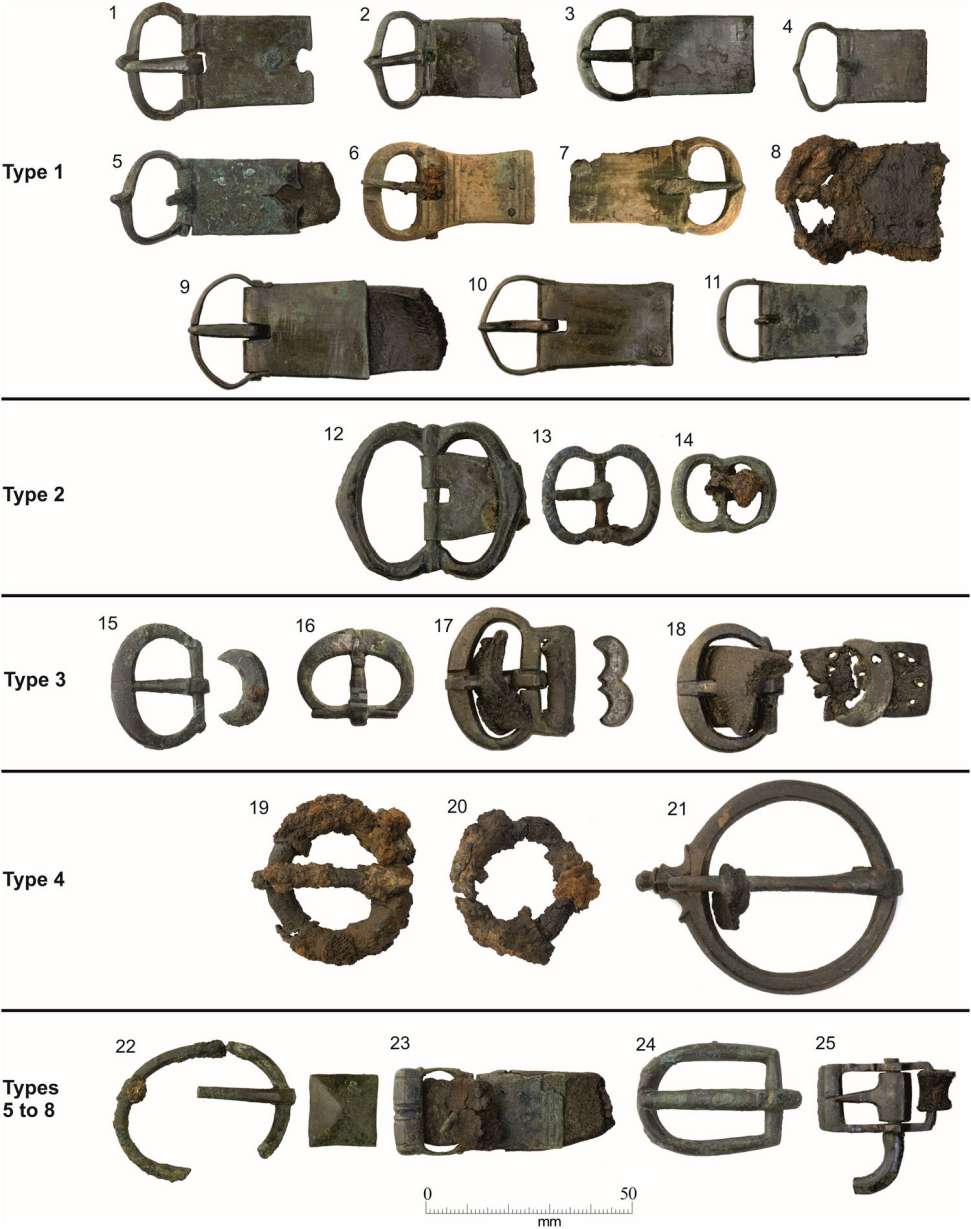
Buckles from the Cambridge Augustinian friary, copper alloy unless otherwise stated. (1) Type 1, burial F.216. (2) Type 1, burial F.265. (3) Type 1, burial F.334. (4) Type 1, burial F.336. (5) Type 1, burial F.352. (6) Type 1, animal bone, burial F.347. (7) Type 1, elephant ivory, F.314. (8) Type 1, iron, burial F.348. (9) Type 1, 1908–9 discovery Z 1923.1597 B. (10) Type 1, 1908–9 discovery Z 1923.1597 C. (11) Type 1, 1908–9 discovery 1910.273. (12) Type 2, burial F.191. (13) Type 2, burial F.230. (14) Type 2, burial F.310. (15) Type 3A, burial F.331. (16) Type 3A, burial F.332. (17) Type 3B, 1908–9 discovery 1910.275 B. (18) Type 3A, 1908–9 discovery 1910.271. (19) Type 4, iron, burial F.311. (20) Type 4, iron, burial F.333. (21) Type 4 1910.270. (22) Type 5 with mount, burial F.302. (23) Type 6, burial F.367. (24) Type 7, burial F.344. (25) Type 8, 1908–9 discovery 1910.274. *Photographs by Dave Webb, © Cambridge Archaeological Unit. Images of buckles discovered in 1908–9 courtesy of the Museum of Archaeology and Anthropology, University of Cambridge.*

**Fig 9:**
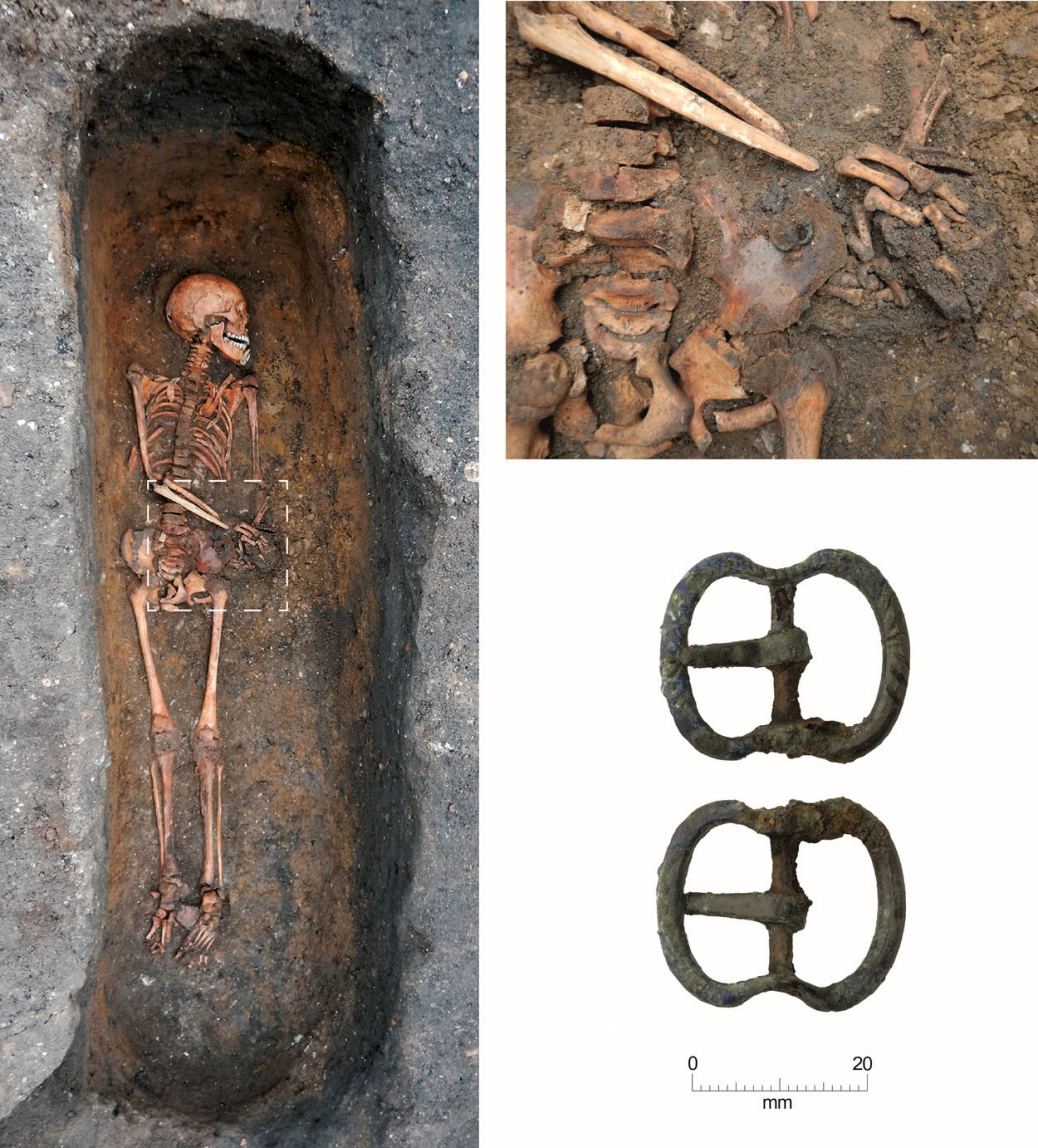
Cambridge Augustinian friary, chapter house, burial F.230 plus in situ detail of buckle. Type 2 copper alloy buckle pointing right. *Photographs by Craig Cessford and Dave Webb, © Cambridge Archaeological Unit.*

There are eight buckle forms that accompany burials at the Cambridge Augustinian friary (Fig 8).^53^ Only four forms are represented by more than a single buckle. The most common form Type 1, a D-shaped frame with a rectangular plate (11 examples), can be interpreted as the ‘default’ buckle form of the late 13th–mid-14th-century friary. One of the other common forms Type 2, with a symmetrical double oval frame (three examples), may have fulfilled the same role at a later date in the mid-/late 15th–mid-16th centuries and is restricted to the chapter house (Fig 9; see also Fig 5).

**Fig 10:**
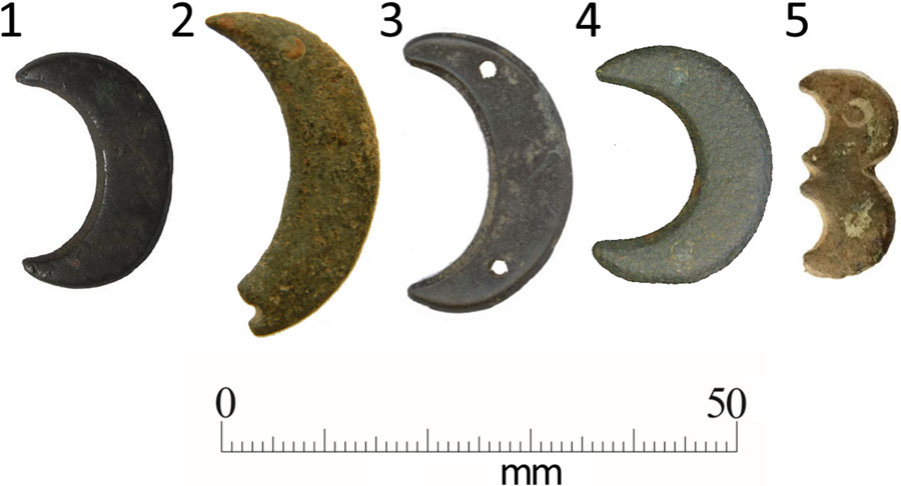
Examples of copper alloy crescent and double-crescent mounts with pairs of rivets recorded through the Portable Antiquities Scheme. (1) Crescent mount from Addlethorpe, Lincolnshire, LIN-011E76^48^, (2) Crescent mount from Quarley, Hampshire, WILT-6C6516^49^, (3) Crescent mount from Roundway, Wiltshire, WILT-4B3FC7^50^, (4) Crescent mount from Beeston with Bittering, Norfolk, NMS-419B53^51^, (5) Double-crescent mount from Brayton, North Yorkshire, SWYOR-B37CC6^52^, *Reproduced through a CC BY-SA 4.0 licence. Rights holders (1) Lincolnshire County Council, (2) Salisbury and South Wiltshire Museum, (3) Wiltshire Archaeological and Natural History Society, (4) Norfolk County Council and (5) West Yorkshire Archaeology Advisory Service.*

Type 3 with oval or D-shaped buckle frames and crescent or double-crescent mounts (four examples), appears to be an unusual type. One possibility is that although there is some overlap, Type 3 largely fall between Types 1 and 2 chronologically. Crescent and double-crescent mounts are rare in England. In a survey of over 750 examples there are none of these forms and the common types are circular (251), foliate (178), bar (175) and rectangular (71).^54^ Similar plain crescent and double-crescent mounts with pairs of rivets are known from the Portable Antiquities Scheme, with at least four similar single crescent mounts from Hampshire, Lincolnshire, Norfolk and Wiltshire, and a double-crescent mount from North Yorkshire (Fig 10). This demonstrates that their use was geographically quite widespread and makes it likely that their use was not restricted to Augustinian friars. Buckles with crescent and double-crescent mounts have, however, also been found at the Hull Augustinian friary (see below), suggesting that they may have been particularly favoured by the Order.

Crescents were already in use as a religious symbol in England prior to the arrival of the Augustinian friars. A general chapter of the Augustinian canons at Newburgh in 1247 specifically prohibited the use of horse harness mounts (*phaleras equorum*) in the form of a small, crescent-shaped moon (*lunulis minutim*).^55^ While the crescent could have had a range of symbolic meanings, for Augustinians the most likely explanation is that it is inspired by the works of St Augustine himself. In his Expositions on the Book of Psalms (*Enarrationes in psalmos*), Augustine refers to the crescent moon as a symbol for the Church and states that there are two opinions of the meaning of this.^56^ The first is that the waxing and waning of the moon represents a spiritual/human duality, while the second is that the moon shines due to reflected light from the sun and this represents the relationship between the Church and Jesus. The crescent would therefore be an appropriate Christian symbol, which was particularly apposite for the friars given the link to the writings of St Augustine.

**Fig 11:**
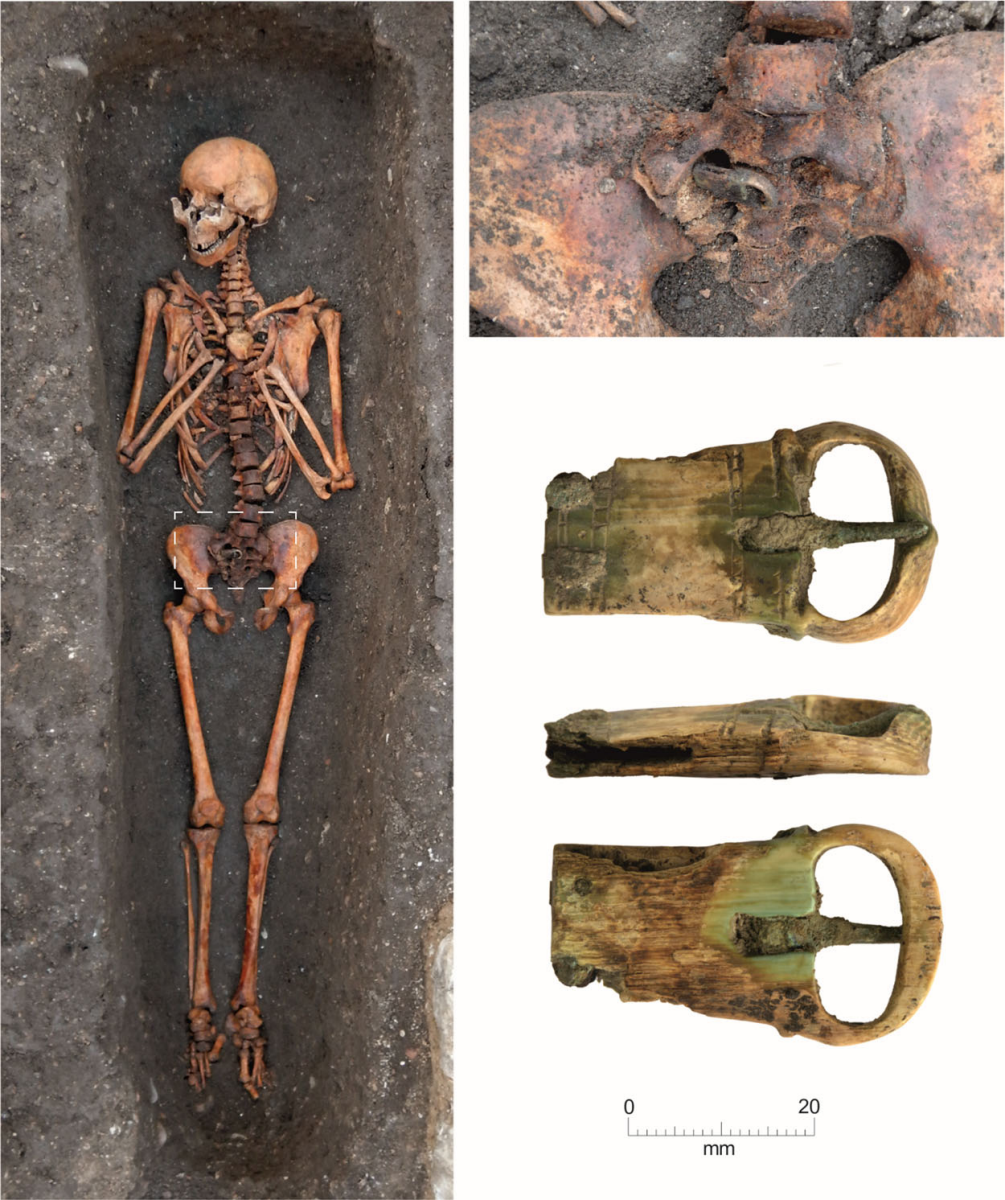
Cambridge Augustinian friary cemetery, burial F.314 plus in situ detail of buckle. Type 1 elephant-ivory buckle, pointing left. *Photographs by Craig Cessford and Dave Webb, © Cambridge Archaeological Unit.*

The other examples (Types 4–7; six examples) indicate that, although there may have been a series of ‘default’ buckle forms at the friary, other buckles were in use. Given that there was significant movement of friars between friaries one possibility is that the Type 4–7 buckles were brought to Cambridge as part of the clothing of friars from elsewhere. It is also tempting to imagine that the bone and ivory buckles, neither of which are likely to have been manufactured locally, travelled as parts of friars’ clothing. The ivory buckle is probably of French origin, and is likely to have been manufactured in Paris, as this was the major centre of production (Fig 11).^57^ This buckle represents a relatively exclusive product requiring considerable individual craftsmanship compared to the mass-produced metal buckles. In contrast, the bone buckle may well have been made in England, quite possibly in northern England, as there are parallels from Goltho (Lincolnshire) and York.^58^ French students appear not to have studied at Cambridge (see Fig 1), presumably because of the pre-eminence of the Paris *studium generale*. There are, however, documented instances of English friars studying in Paris and returning to England after a few years.^59^ The earliest evidence for English Augustinian friars studying in Paris is in 1303, when Rudolphus and Galterus de Anglia (Rudolph and Walter of England) were amongst a number of foreigners at the *Quai des Grands Augustins studium generale*.^60^ As the ivory buckle was associated with the burial of an individual who died aged 18–25 in c 1320–49, it is possible that they were an English friar who had studied in Paris and then returned to England. Additionally, travel to Rome where the Prior General of the Order was based would often have been via Paris. It is tempting to suggest that the ivory buckle could have been a visible symbol indicating that a friar had studied in Paris; however, the broadly contemporary bone buckle associated with a friar buried c 1300–49 would have been visually indistinguishable. It is also, however, conceivable that all these buckles were purchased in Cambridge.

**Fig 12:**
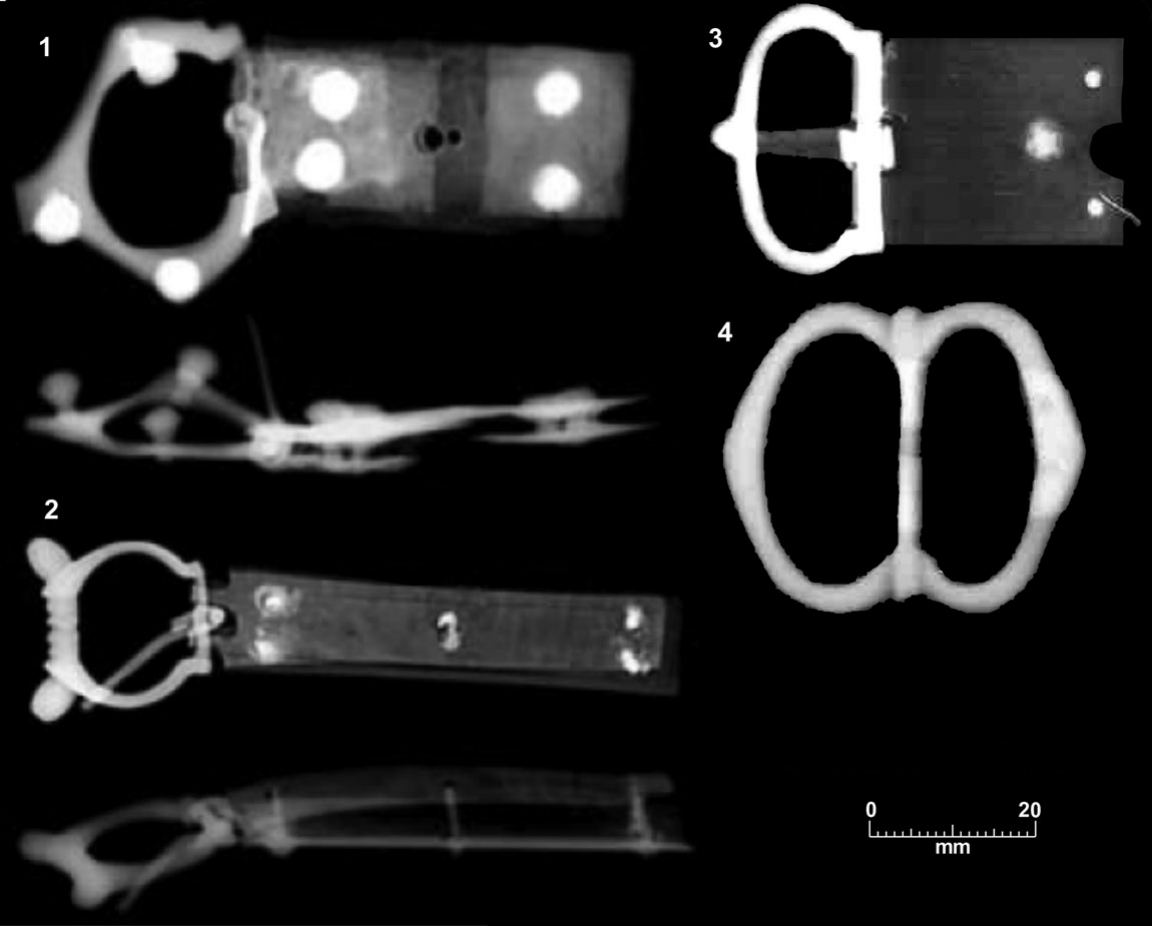
X-rays of 14th-century copper alloy buckles. (1–2) 14th-century buckles from nearby domestic site at Hostel Yard, Corpus Christi College. (3) Type 1 buckle from Cambridge Augustinian friary cemetery burial F.216. (4) Type 2 buckle from Cambridge Augustinian friary chapter house burial F.191. *X-rays by the York Archaeological Trust for the Cambridge Archaeological Unit*.

The buckle forms present at the friary are all relatively plain, which fits well with mendicant ideals of poverty and Augustinian views on dress. At most, they have a single functional mount, with no evidence for the complex belt sets with multiple mounts and other features that are known from other cemeteries.^61^ In contrast ‘Among ordinary people of both sexes, the opportunity for self-expression lay in the wide variety of mounts and fittings that could be applied to leather and textile girdles, including rivets, spangles, loops, eyelets and strap-ends’.^62^ Although relatively few buckles have been recovered from other archaeological contexts in Cambridge, it is notable that many of these, such as two from the nearby site of Hostel Yard, are noticeably larger and more ornate than those from the friary (Fig 12).^63^ Some of the forms represented at the friary have been recovered from excavations on domestic sites in Cambridge, and more broadly are recorded by the Portable Antiquities Scheme from locations in Cambridgeshire. Buckles with D-shaped frames and rectangular plates (Type 1) were in use in Cambridge and Cambridgeshire, but whereas they represent 44% of the buckles from the friary they are fewer than 10% of the buckles from both Cambridge and Cambridgeshire. Buckles with symmetrical double oval frames (Type 2) are relatively common from Cambridge and Cambridgeshire, but crescent-shaped mounts (Type 3) and bone or ivory buckles are otherwise unknown from Cambridge and Cambridgeshire.

The black background of the Augustinian habit and girdle would have provided an excellent contrast for the buckles. In their original untarnished golden state, copper alloy buckles would also have been highly visible. The white bone and ivory buckles would have been even more visible, which may explain the presence of these rarer materials that required much greater individual work and artisanship than the mass-produced copper alloy buckle.^64^ The iron buckles would probably have been the least distinctive, even in their original silver-coloured state.

## BUCKLE ORIENTATION

The buckles still appear to be in the position where they originally lay in the grave, although a small amount of post-depositional movement is possible. Most of the buckles were orientated in a horizontal position relative to the skeleton. They point either to the right side of the deceased (16 instances; for a typical example see Fig 6) or to the left (one instance; see Fig 11). Cross-sectional geometry was used to measure bilateral asymmetry of bone structure and determine which arm of the skeleton displayed adaption to greater habitual loading.^65^ Although directional asymmetry does not necessarily equate to handedness, there is generally a good correspondence between hand preference and side dominance in cross-sectional properties.^66^

**Fig 13:**
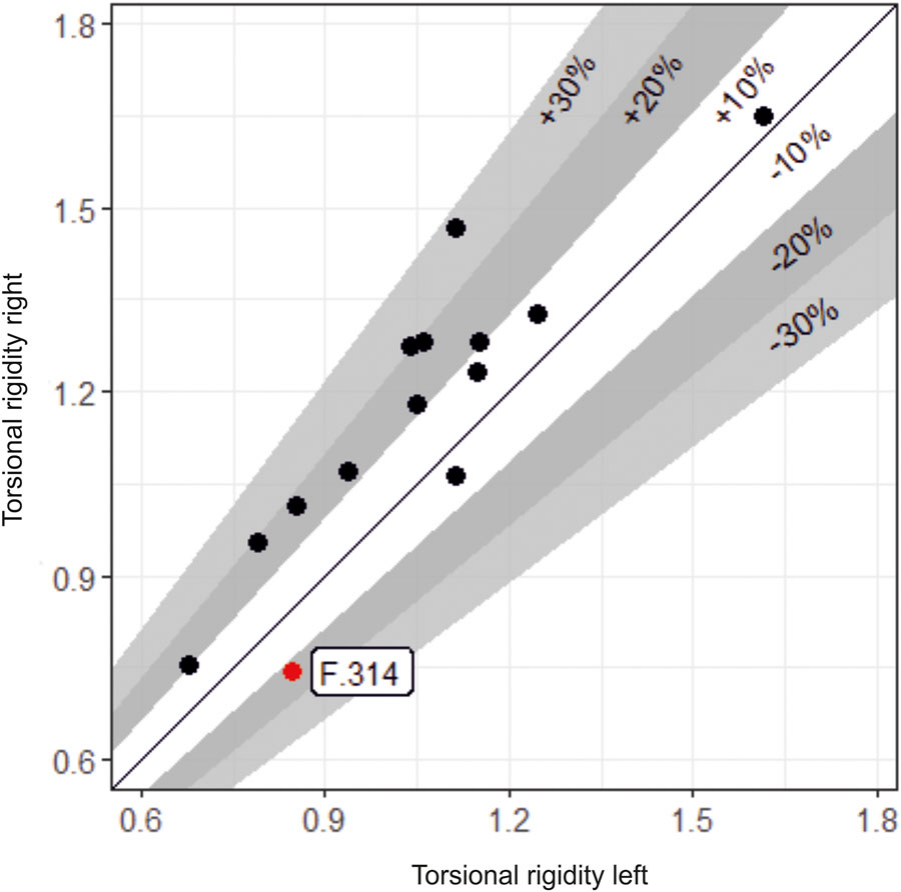
Bilateral asymmetry plot. Humeral bone structure in individuals from the Cambridge Augustinian friary with associated buckles. *Diagram by Bram Mulder.*

Fourteen individuals with associated buckles were measured; 12 (86%) of these favoured the right and only two the left (14%). Although cross-sectional geometry and handedness should not be simplistically equated, this broadly matches estimates that c 90% of the population are right handed. The only individual with the buckle pointing to the left (F.314; Fig 13) displayed the greatest left-directional asymmetry (13%). The other individual with a left-side dominance (F.334) had an asymmetry of 5% and their buckle pointed right. It appears that F.314 was left-hand dominant and this is reflected in how their girdle was fastened after death.

There are elements of the Bible that favour the right side over the left, notably the righteous/sheep being set on the right and the fallen/goats on the left (Matthew 25: 32–3). There is evidence for prejudice in the medieval period and attempts to make left-hand dominant individuals use their right hands, although this does not appear to have been systematic. Writing would have been easier for a right-hand dominant scribe, there is however evidence for works by left-hand dominant scribes.^67^ The evidence from the Cambridge friary indicates that it was possible for a friar to be left-hand dominant.

**Fig 14:**
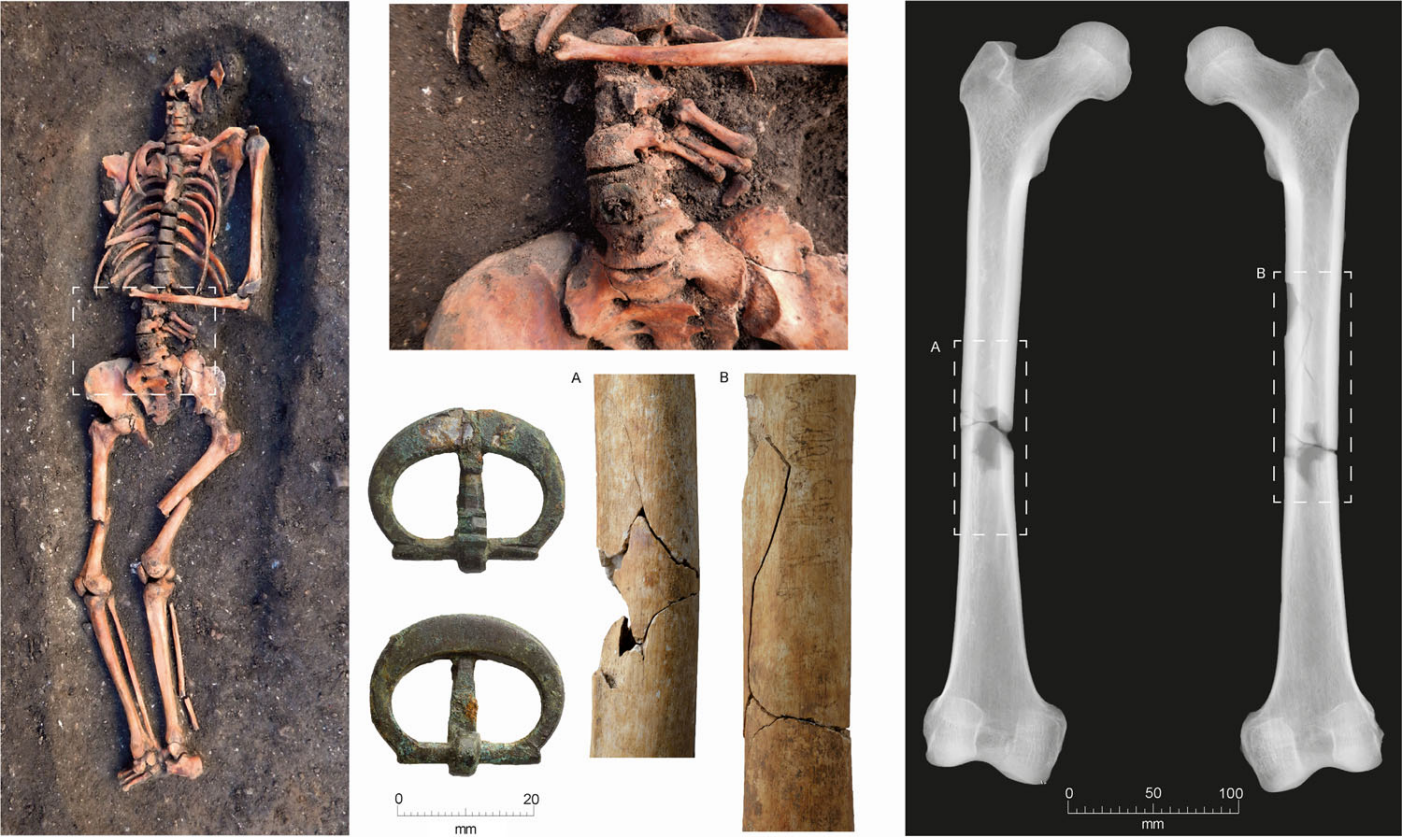
Cambridge Augustinian friary cemetery, burial F.332. Showing Type 3A copper alloy buckle lacking crescent-shaped mount, pointing vertically upwards. Also X-ray and photographs of leg fractures. *Photographs by Craig Cessford and Dave Webb, © Cambridge Archaeological Unit*. *X-ray by Reveal Imaging for the After the Plague project.*

In a single case, the buckle is aligned vertically relative to the skeleton, pointing upwards (F.332: Fig 14). This individual died from a major accident, which resulted in bilateral femoral comminuted butterfly fractures. They also had fractured vertebrae at the cervicothoracic junction, possibly from a whiplash-type injury likely to be concordant with the femoral fractures. Although technically these injuries are survivable, it is likely that major arteries were severed leading to death from haemorrhagic shock within a few minutes. Even if this were not the case, death would probably take place within a few weeks from infection. The girdle also appears to have been damaged, as it is missing its associated mount. One interpretation is that the buckle was damaged in the accident that killed the individual and this meant that the girdle had to be fastened in an unorthodox manner, perhaps by being tied in a loop. Alternatively, the girdle may have been folded and laid on top of the body with the buckle facing vertically. The left and upwards facing buckles suggest that the girdles that individuals were buried with were those they used in life.

## CLOTHED BURIAL

It is often assumed that from the 8th century onwards most British burials were shrouded, although shrouds rarely survive and shroud pins are uncommon, and their use is largely inferred from body position, and textual and artistic evidence.^68^ During the 11th–12th centuries the clergy and members of religious orders came to be distinguished by clothed burial, in some cases also being accompanied by artefacts such as chalices and patens and croziers as expressions of a distinct form of religious masculinity.^69^ Religious clothing did not simply represent the identity or status of the deceased, it was intended to protect the physical body of the corpse in purgatory and hasten salvation. Clothed burial also reinforced distinctions; between clerics and laity, between different religious orders, and within religious hierarchies.

After a member of the Augustinians died, there would be prayers and the corpse would be washed and then clothed in the distinctive habit and girdle of the Order.^70^ One pertinent issue is whether the clothing that an individual was buried in was that which they wore in life, especially as under the Rule of St Augustine clothing was communally owned and stored.^71^ The buckles from Cambridge all show signs of use and wear, with some being repaired, indicating that these were not items specifically produced for use after death. This does not, however, necessarily indicate that the buckles/girdles were particularly associated with the same individual in life and death.

## CLOTHED BURIAL AT OTHER SITES

### Clothed Burial Elsewhere in Cambridge

There is no evidence that clothed burial was particularly common at other burial grounds in late-medieval Cambridge. Of the excavated broadly contemporary cemeteries in and around Cambridge, several have not produced any evidence for dress fittings (Tab 2). In others, the frequency does not exceed 1.5%, even when allowance is made for factors such as truncation. Although there are few dress fittings at any of these sites, where they occur they are characterised by greater variety than at the Augustinian friary.

**Table 2:** Dress fittings as evidence for clothed burials in late-Saxon to late-medieval cemeteries in and around Cambridge.

Site	Cemetery type	Date	No burials	No clothed burials	% clothed burials	Reference
All Saints by the Castle, Cambridge	Urban, parochial	Mid-10th–1365	213	0	0	Cessford et al, in prep
St Bene’t’s, Cambridge	Urban, parochial	11th–mid 14th	35	0	0	Cessford 2005
St John the Evangelist, Cambridge	Urban, hospital	1204–1511	400	1	0.25 * c 0.4	Cessford 2015
Church End, Cherry Hinton	Rural, proprietary	Mid-10th–mid 12th	670	6	0.90 *c 1.2	Lally 2008
Clopton	Rural, parochial	13th–mid 16th	83	0	0	Alexander 1968
Crowland Road, Haverhill	Semi-urban, parochial	10th/12th–14th	355	4	1.13 *c 1.5	Murray 2005

*Allowance made for truncated burials etc.

### Clothed Burial at Other Augustinian Friaries in England

Although excavations have taken place at a number of other English Augustinian friaries, only a few have revealed significant numbers of burials. One complicating factor is that burial at friaries was polyfocal, with different groups tending to be buried in different locations. These included the church, cloister walk and garth, chapter house and cemeteries. This means that it is problematic to compare excavations, as they are often of different parts of friaries and therefore of different burial populations. For the Cambridge evidence, the most useful parallels are the Augustinian friaries at Leicester and Hull (Tabs 3–4).^72^

At the friary in Leicester (1254–1538), clothed burial appears to have been relatively common. Eight of the 26 burials (31%) had copper alloy (five) or iron (three) buckles, which accompanied male, female and non-adult (defined as aged under 18) individuals and it has been argued that lay individuals may have been buried wearing religious clothing.^73^ Although non-adult and ‘female’ skeletons had associated buckles, it is possible that all these individuals were in fact members of the Order. The non-adults were aged 11–15, making them old enough to have been novices, while the two ‘female’ skeletons are only ‘probably’ female and could be male. Most of these buckles were lying on the pelvis and slightly to one side, indicating a girdle worn low over the hips. The Leicester buckles include examples of the Cambridge Type 1 (three examples), Type 2 (one example), Type 4 (one example) and Type 5 (one example). The Leicester girdles display more variation than Cambridge, with three different types: wide girdles with decorative slashing/stitching, girdles with stud impressions and narrow girdles.^74^ One of these girdles associated with a ‘woman’ was only 14 mm wide, while the others were 20+ mm and the wide girdles were 38–50 mm.

At the Hull Augustinian friary (1316/17–1539), 255 burials were excavated; including 39 individuals with buckles of copper alloy (34) and iron (5).^75^ The areas investigated were rather different from those at Cambridge and there is evidence for a much wider range of clothing, some of it definitely associated with members of the laity. There is also evidence for clothed burials lacking any metal dress accessories.

The individuals with buckles were predominantly male, although five buckles were found with possible females, and one iron buckle associated with a definite female had a separate small coffin containing a perinatal infant laid over one of the legs. There were nine non-adults with buckles, the youngest two individuals aged between six and ten. Although these are too young to be novices, both are rather unusual. One comes from a mass burial of eight individuals, where they and two of the adults both had dress accessories. The other had a buckle, strap end, three cinquefoil mounts and a lace end. There is good evidence that at the Hull friary clothed burial was not solely limited to members of the Order so these two non-adults may well be lay individuals. Similarly, an individual in a coffin with two breche buckles as opposed to a girdle buckle may also be laity. Most of the buckles are, however, single girdle buckles and the association of most of the buckles with males and their rather uniform nature and restrained decoration suggests that they may be linked to religious members of the friary.^76^

Burials with buckles were widely distributed, being found in the church presbytery and nave and cloister walk and garth (Tab 4). Overall, 11% of skeletons had buckles, although this may well be an under-representation due to truncation. In ‘almost all’ instances the buckles were near the pelvis or waist of the skeleton and the high number of instances was deemed as being incompatible with them being priests’ burials.^77^ The copper alloy buckles are not typical of the normal range from sites of the period, many common types are absent and there is a restricted range of forms, these are not particularly rare types but neither are they the most common.^78^

**Table 4:** Evidence for burials with girdle buckles at the Cambridge, Hull and Leicester Augustinian friaries.

Friary	Location	Total	Adult	Adult male	Adult female	Non-adult	Girdle buckles	Skeletons associated with belts
Cambridge	Cemetery	32	27	27	0	5	16 50%	15 adult males, 1 non-adult male
Cambridge	Chapter house	6	4	3	1	2	4 67%	3 adult males, 1 non-adult male
Cambridge	Cloister: all	48	46	42	4	2	7 15%	Associations unknown, probably mainly adult male
***Cambridge***	***Total***	***86***	***77***	***72***	***5***	***9***	***27*** ***31%***	***18 adult males, 2 non-adult males***
Hull	Church: nave	152	136	80	39	12	10–11 7–8%	8–9 adult males, 2 adult possible females
Hull	Church: presbytery	46	43	32	8	2	10–12 23–28%	7–9 adult males, 3 adult possible males
Hull	Church: all	198	192	112	47	14	20–23 10–12%	15–18 adult males, 3 adult possible males, 2 adult possible females
Hull	Cloister: garth	3	3	1	2	0	0	None
Hull	Cloister: walk	49	33	27	6	15	7 14%	5 adult males, 2 adult possible males
Hull	Cloister: all	52	36	28	8	15	7 13%	5 adult males, 2 adult possible males
Hull	Unknown	5	5	3	1	0	0	None
***Hull***	***Total***	***255***	***233***	***143***	***56***	***29***	***27–30*** ***11–12%***	***20–23 adult males, 5 adult possible males, 2 adult possible females***
Leicester	Chapter house	6	2	2	0	4	1 17%	1 adult male
Leicester	Cloister: garth	2	2	2	0	0	1 50%	1 adult male
Leicester	Cloister: walk	10	7	6	1	3	5 50%	3 adult males, 1 adult possible female, 2 non-adults
Leicester	Cloister: all	12	9	8	1	3	7 58%	5 adult males, 1 adult possible female, 2 non-adults
Leicester	Southern cemetery	8	7	7	0	1	0	None
*Leicester*	*Total*	*26*	*18*	*17*	*1*	*8*	*8* *31%*	*6 adult males, 1 adult possible female, 2 non-adults*
**All**	**Total**	**367**	**328**	**232**	**62**	**46**	**62–65** **17–18%**	**44–47 adult males, 6 adult possible males, 3 adult possible females, 4 non-adults**

Ten of the buckles had single or double crescent-shaped mounts, which closely parallel the Type 3 buckles from Cambridge.^79^ Asymmetrical double-looped buckles were common at the Hull Augustinian friary (eight associated with burials plus three others) and there is some evidence that they may be more particularly common in religious contexts.^80^ These are similar to the Type 2 buckles from the Cambridge friary, although those were symmetrical.

Dress accessories are less well represented at other Augustinian friary excavations, detailed information is not available for Winchester although it appears that buckles may have been relatively common (Tab 3). The single burials with buckles from Canterbury and Warrington appear similar to those from Cambridge (Tab 3). At Warrington the investigations focussed on the nave of the church and it is likely that, ‘these individuals represent lay patrons and their families rather than friars’.^81^ In general, the lower representation of buckles at some friaries may in part be because the proportion of members of Augustinians to laity buried in the investigated areas of these friaries was lower.

**Table 3:** Dress fittings as evidence for clothed burials in selected English late-medieval Augustinian friaries and those of other orders where numerous burials have been excavated.

Site	Order	No burials	No clothed burials	% clothed burials	Comments	Reference
Cambridge, 2016–17	Augustinian	38	20	52.6		This article
Cambridge 1908–9	Augustinian	48+	7+	23.5	May be an under-representation	This article
Hull	Augustinian	255	39	15.3	See text	Evans in prep; Gilchrist and Sloane 2005 b
Leicester	Augustinian	26	8	30.8	See text	Mellor and Pearce 1981
Warrington	Augustinian	30	1	3.3	Plain D-shaped or oval copper alloy girdle buckle	Heawood 2002
Canterbury	Augustinian	52	1	1.9	D-shaped copper alloy girdle buckle	Hicks 2015, 26–7
Winchester	Augustinian	Unknown	Unknown	Unknown	Buckles found with several skeletons	O’Sullivan 2013, 341
Aberdeen	Carmelite	201	1	0.5	Copper alloy bracelet on left wrist	Cameron et al 2019
Carlisle	Dominican	214	1	0.5	Double buckle	McCarthy 1990, 77–80
Norwich	Franciscan	136	0	0.0		Soden 2010
Carmarthen	Franciscan	260	0	0.0		James 1997

Figures in brackets indicate allowance made for truncated burials.

The buckles from both the Leicester and particularly Hull friaries show distinct parallels with those from Cambridge. In England, the 35 Augustinian friaries of the national province were organised into four limits or districts. Cambridge was the head house of a limit that broadly covered East Anglia, including seven other houses at Clare, Huntingdon, Little Yarmouth (Gorleston), King’s Lynn, Norwich, Orford and Thetford. The Leicester friary was one of the 11 houses of the Lincoln limit, while the Hull friary was one of the seven houses of the York limit. Annually, each limit could send up to four friars to study in the Cambridge *studium*, and contacts between Cambridge and friaries in other limits are to be expected.^82^ As the clothing they were wearing, including girdles and their associated buckles, was one of the few items that friars would take with them while travelling between friaries a degree of movement and commonality of material culture between Augustinian friaries in the national province is inherently plausible.

### Clothed Burial and Other Religious Orders in England

A survey of late-medieval dress accessories concluded that it was common for clergy to be buried in their daily clothes of office and that these did not use the numbers of accessories that would have been in use in contemporary secular fashions.^83^ The evidence suggests they wore leather girdles with a single or double buckle, either to secure the outer habit or underneath it supporting the breeches and hose. The survey included 22 buckles found directly associated with burials, of which 13 were Augustinian.^84^ While some accessories seem to contravene the regulation of religious life, many show evidence of reuse and repair, namely on buckles with replaced pins. This suggests a frugal lifestyle consistent with the rules of the various orders.^85^ A comparison between religious sites and assemblages from waterfront dumps in London indicates that buckles are more prominent at religious sites and mounts less common.^86^ At York, a comparison between the religious sites of the College of the Vicars Choral and the Gilbertine priory indicates that for the Gilbertines simplicity in dress was an expression of their withdrawal from ‘normal’ society, signalling their otherness.^87^ Conversely, the members of the Vicars Choral were actively choosing to use more elaborate forms of dress accessory, as visual displays of their social position within society, resulting in a much wider and more decorative range of dress accessories.^88^

The three other main orders of friars in England were the Franciscans, Dominicans and Carmelites, who were all distinguished by different clothing. Although leather girdles were particularly associated with the Augustinians, they were also worn by Dominicans, and Carmelites. In contrast, the Franciscans used simple white ropes or cords known as *cinctures*, with three knots symbolising poverty, chastity and obedience. Where large groups of skeletons related to the other orders have been investigated, a much lower proportion of burials have associated dress accessories, and these are more disparate and apparently largely related to the laity (Tab 3). The contrast with the Franciscans who did not wear leather girdles and espoused a much greater commitment to absolute poverty is particularly revealing.^89^ At the Carmarthen (Wales) Franciscan friary where over 200 burials were excavated, there appear to have been no metal dress accessories.^90^ The same is true for Norwich, where 136 individuals were excavated and the high proportion of sub-adults and young adults has been interpreted as linked to the presence of a high proportion of postulant friars and scholars at the *studium*, but no dress accessories were found.^91^

## DISCUSSION

Late-medieval buckles are archaeologically common and were originally closely associated with the specific individuals who wore them. Despite this, in the archaeological literature buckles have usually become generic and anonymous. Detailed contextualisation of the buckles associated with skeletons at the Cambridge Augustinian friary readdresses this in an unparalleled manner for late-medieval British archaeology. This transforms the functional and symbolic role of the buckle, linking these objects to corporate and personal identity.

Although clothing was important to medieval Augustinian friars, the textual sources do not mention the girdle buckles that survive archaeologically. The girdle buckles from Cambridge indicate that many of the individuals there were buried clothed, this clothing included a leather girdle and almost certainly denotes membership of the Augustinian Order. No other dress accessories were present, and these individuals were presumably buried in woollen habits and with shoes that lacked metal buckles. The buckles are relatively small and simple, in keeping with mendicant ideals of poverty, although the widths of the leather girdles do not correspond to the dimensions prescribed for the Order. The other individuals buried at the site were probably lay benefactors and servants, who were shrouded rather than clothed. The high proportion of burials with girdle buckles and the lack of evidence for other dress fittings contrasts strongly with most other late-medieval cemeteries in England. The closest parallels come from the other Augustinian friaries at Hull and Leicester, although even at these sites the proportion of burials with girdle buckles was lower. There is more evidence for variation in dress accessories at Hull. At least some clothed individuals were members of the laity, and it is possible that also took place in Cambridge in areas such as the church that have not yet been archaeologically investigated.

Although there is some variation in the form and material of the buckles from the Cambridge friary, the dominance of a relatively restricted range of forms suggests that the buckles were generally supplied by the Order. The presence of a range of other buckle forms may suggest that some were acquired by other means, perhaps representing charitable gifts, acquisitions by individual friars or items brought by individuals travelling from other friaries. There is also evidence suggesting that the girdles individuals were buried with were probably those they wore in life. The evidence from the Hull and, to a lesser extent, Leicester friaries suggests that there was a degree of shared material culture through the national province of Augustinians, including buckles with unusual crescent-shaped mounts whose symbolism is probably linked to the writings of St Augustine. An unusual ivory buckle potentially links the study centres of Paris and Cambridge, emphasising the international profile and connectivity of the Order.

## Supplementary Material

Supplemental Material

## Footnotes

9 Gilchrist 2012, 74, 81; Egan, in Bowsher et al 2007, 349.

10 Egan and Pritchard 1991.

11 Ibid.

12 Schofield et al 2018, 308–10.

13 Eg Biddle 1990; Margeson 1993; Ottaway and Rogers 2002.

14
*https://finds.org.uk/*

15 Cassells 2013; Smith 2009; Standley 2010; 2013.

16 Eg Cassels 2013.

17 Brown 2018; Jeffries 2012; Watson 2015a; 2015b.

18 Gilchrist and Sloane 2005a, 80–7.

19 The clothing of other contemporary religious orders in Britain lies beyond the scope of this paper, but important works include Fizzard 2007; Harvey 1988; Oldland 2013.

20 Concha Sahli 2017, 106–78; 2018; Warr 2007.

21 Cendoya 1966.

22 Arbesmann and Hűmpfner 1943; Rotelle 1993, 48–57.

23 Concha Sahli 2018, 114.

24 Cessford and Neil in prep.

25 Cessford et al 2021.

26 Laferrière 2017, 41–2, 150–1, 247, 273.

27 Ibid 247.

28 Ibid 86.

29 Lawless 1987, 87.

30 Lawrence 2013.

31 Roth 1966 vol **I**, 250.

32 Roth 1966 vol **II**, 54–5, no 97.

33 Ellis and Salzman 1948, 288.

34 Cessford 2017; Cessford and Neil in prep.

35 Cranage and Stokes 1921; Duckworth and Pocock 1910.

36 Spyrou et al 2019; see also Cessford et al 2021.

37 Supplementary Material, Appendix 1.

38 Gilchrist and Sloane 2005a, 85–7, tab 3.

39 Gutiérrez 1984, vol **I**, 63.

40 Roth 1966 vol **I**, 136–7.

41 Burrow 1988, 80–90; Sears 1986, 55–62.

42 Lawrence 1984, 217; Mays 2009, 182.

43 Supplementary Material, Appendices 2 and 3.

44 Supplementary Material, Appendix 4.

45 Supplementary Material Appendix 5.

46 Egan and Pritchard 1991, 21, tab 1.

47 Cassells 2013; Quita Mould, pers. obs.

48 https://finds.org.uk/database/artefacts/record/id/525598.

49
https://finds.org.uk/database/artefacts/record/id/181339

50 https://finds.org.uk/database/artefacts/record/id/104991.

51
https://finds.org.uk/database/artefacts/record/id/124123

52
https://finds.org.uk/database/artefacts/record/id/974123

53 For typology see Supplementary Material, Appendix 2.

54 Cassells 2013, 71–9.

55 Salter 1922, 31.

56 St Augustine, in Hebgin and Corrigan 1960, 102, 137–40.

57 Stratford 1987, 108–9; Gaborit-Chopin 2003, 21–2.

58 Beresford 1975, 26, 77; Waterman 1959, fig 19.7.

59 Roth 1966, vol **II**, 305–06 no 739, 308 no 747, 431–3 no 1064.

60 Courtenay 2001, 222–3.

61 Gilchrist and Sloane 2005a, 84–5.

62 Gilchrist 2012, 74.

63 Cessford 2005.

64 In the 1690s, Gabriel d'Emiliane (c 1655–1714), a French secular Roman Catholic priest who became a protestant refugee, was living in London and had taken Anglican orders. He published a history of monastic orders, which stated that Pope Alexander IV gave the Augustinians ‘the pretended Rules of St Augustine, joined them in a body under one general, ordering them to wear the same habit, to wit, a long gown with broad sleeves, a fine cloth hood, and under these black garments other white ones, and that they should tie about their middle a leathern girdle, fastened with an ivory bone’ (d'Emiliane 1693, 48). Richard Newcourt repeated this statement (Newcourt 1708 vol **I**, 289), and was himself widely repeated, but the basis for the ‘ivory bone’ girdle fastening is unknown.

65 Mulder 2020.

66 Lazenby 2002; Shaw 2011; Stock et al 2013

67 Parkes 2008, 62–3.

68 Mui 2015.

69 Gilchrist 2009; 2012.

70 Andrews 2006, 132. The process for other religious orders was similar, for a detailed description of Cluniac practice see Williams 2015, 56; 2018.

71 Lawless 1987, 75, 81, 87, 93, 95.

72 Evans forthcoming; Mellor and Pearce 1981

73 Clay 1981, 133, 135, 137–9; Daniell 1997, 136.

74 Allin 1981, 158–60.

75 Data from Gilchrist and Sloane 2005b.

76 Goodall, A forthcoming; Goodall, I forthcoming.

77 Ibid.

78 Gilchrist and Sloane 2005a, 84–85; Goodall, A forthcoming.

79 Goodall, A forthcoming.

80 Ibid.

81 Heawood 2002, 178.

82 Roth 1966 vol **I**, 153.

83 Standley 2010; 2013.

84 Standley 2010, 165.

85 Ibid, 51.

86 Ibid, 106.

87 Cassels 2013, 152–4.

88 Standley 2010, 195.

89 Lambert 1961; Robson 2006.

90 James 1997.

91 Soden 2010.

92 For further detail on skeletons see Neil in Cessford 2017 and Cessford and Neil in prep.
