# Supplementary material for: Buried with their Buckles On: Clothed Burial at the Augustinian Friary, Cambridge
Source: Mediev Archaeol. 2022 Jun 13;66(1):151–87. doi: 10.1080/00766097.2022.2065066 (PMC9197221; doi:10.1080/00766097.2022.2065066)
Supplement: Supplemental Material [file YMED_A_2065066_SM5325.docx]

# Buried with their Buckles On: Clothed Burial at the Augustinian Friary, Cambridge

# Supplementary Material

## APPENDIX 1: DATING OF INDIVIDUAL BURIALS

## *By* Craig Cessford

The dating of individual skeletons was based on a number of criteria. All skeletons are assumed to post-date the acquisition of burial rights by the friary in 1290 and to pre-date the Dissolution of the friary in 1538. All skeletons of women, individuals that are too young to have belonged to the Augustinians (under 14 years old in the cemetery and under 11 years in the chapter house) are assumed to post-date the extension of burial rights to include individuals who were not members of the community in 1302.

In broad terms, the cemetery stratigraphically pre-dates the construction of the cloister. Typological dating of architectural fragments from the cloister arcade indicates that it was constructed after c 1330. It would typically be dated to c 1330–50, although a date as late as c 1390 is feasible. Given the evidence that the cemetery continued in use for a period after the skeleton that tested positive for *Yersinia pestis* this part of the cloister cannot have been constructed prior to c 1360 and more probably c 1370. Bayesian modelling of three radiocarbon determinations from a stratigraphic sequence of skeletons in the cemetery indicates that there is only a 3% likelihood that the cemetery went out of use by 1390 and that this should probably be dated to c 1400–40. Other evidence suggests that this is likely to have taken place in the earlier part of this date range c 1400–20. This apparent incompatibility between the architectural typology and the radiocarbon determinations is best explained by the construction of the cloisters being a long-term building campaign that took place over a period of decades. This is supported by archaeological evidence, including the fact that the chapter house was constructed as a freestanding structure before the part of the eastern claustral range that was built over the cemetery. Construction of the cloister is therefore assumed to have begun c 1330–50, probably in the aftermath of the friary acquiring several properties between 1335 and 1338 as prior to this it is unlikely that the friary owned enough of the street block to construct the cloisters. It was probably completed in c 1400–20, having taken place in a number of discrete stages over a period of c 50–90 years. The three groups of burials can therefore be broadly dated as: Cemetery: c 1290–1400/20; Chapter house: c 1330/50–1538; Cloister garth and walk: c 1330/50–1538.

During its existence, the chapter house was modified and the foundations for its internal benches widened. The evidence indicates that this took place around c 1450, burials in the chapter house can therefore be divided into an earlier group broadly dated to c 1330/50–1450 and a later group dated to c 1450–1538.

For the cemetery, the stratigraphic sequence of burials has been taken into account. Where burial cuts have significantly truncated an earlier skeleton, it is conservatively assumed to imply a minimum time gap of 20 years to allow for the decay of soft tissues. Where stratigraphic evidence exists, this indicates that within the individual rows of burials burial progressed from south to north. This appears to have been a general sequence.

The results of radiocarbon dating of individual skeletons, the typological dating of buckles associated with burials and the dating of other items directly associated with skeletons or present in grave fills have also been considered. This evidence has also been taken into account for burials with stratigraphic relationships to these skeletons. All skeletons that stratigraphically pre-date the individual that tested positive for *Yersinia pestis* are dated to 1349 or earlier as the specific aDNA evidence and other factors suggests that the individual died during this plague outbreak.

## APPENDIX 2: BUCKLE TYPES

*By* Craig Cessford, Andrew Hall, Ian Riddler and Justin Wiles

Type 1

The most frequently found form of buckle has a D-shaped frame with a rectangular plate. This is one of the commonest 14th–15th-century buckle types in Britain. They are closest to Egan and Pritchard’s oval frames with rigid composite plates, although some of those from London have oval frames other are more D-shaped and similar to the Cambridge examples.^^[[1]](#footnote-1)^^ There are eleven examples, eight from the cemetery and three from the cloister walk/garth. Although predominantly copper , they include examples in iron (F.348), animal bone (F.347) and elephant ivory (F.314). Most of the buckles have straight attachment ends, but two have grooved apertures (F.216 and F.352). In London, examples with grooved apertures are 14th-century whereas those without are 15th century, but both types appear to be contemporary in Cambridge.^^[[2]](#footnote-2)^^

Type 2

There are three symmetrical double oval frame buckles; all are made of copper alloy and come from burials in the chapter house. Evidence from London indicates that this type of buckle dates to between 1350 and the 16th century, suggesting that they are absent from the cemetery as the burials there are largely too early.^^[[3]](#footnote-3)^^ This may have become a common form of buckle at the friary in the later stages of its existence. These buckles all occur in a distinctive position high up on the pelvis, which is rather different from other buckle types, with two on the lefthand side of the body and one on the righthand side. This may suggest a slightly different dress style.

Type 3

There are four oval or D-shaped buckle frames with crescent (Type 3A) or double-crescent (Type 3B) mounts. All are copper alloy, with two from the cemetery (Type 3A) and two from the cloister walk (Types 3A and 3B). The crescent was missing from one of the buckles from the cemetery, but it is so similar to the other buckle that its existence can be reasonably inferred (F.314, see above). Two buckles from the cemetery and one from the cloister walk/garth correspond to the type with an oval lipped frame with narrowed offset bars, the other example from the cloister walk/garth has an oval lipped frame with a central bar.^^[[4]](#footnote-4)^^ These mounts are functional, as they helped secure the leather girdle and effectively fulfil the same role as a buckle plate. In this they are very different from the multiple decorative mounts of either identical or of differing designs riveted in place along the length of the girdle.^^[[5]](#footnote-5)^^ The two buckles from the cemetery were both stratigraphically late in the cemetery sequence and in the same row, suggesting that the burials are of similar date.

Type 4

Annular buckles, three examples with two iron buckles from the cemetery and one copper-alloy buckle from the cloister walk/garth. Annular buckles are sometimes interpreted as brooches, the position of the iron examples on the skeleton and the presence of a leather strap attached to the copper-alloy example confirm that these were used as buckles. Large annular buckles are particularly common in London on religious sites and accompanying burials and are known from burials at religious sites elsewhere.^^[[6]](#footnote-6)^^

Type 5

Annular buckle with rectangular mount, one copper-alloy example from the cemetery. This buckle was found associated with an individual buried in a small group slightly separate from the main cemetery. All the other burials in this area were truncated and it is unknown if they ever had buckles.

Type 6

Oval frame with ornate outside edge and plate, one copper-alloy example from the cemetery.^^[[7]](#footnote-7)^^ This is a common 14th-century buckle form and occurs in the sequence of burials where iron, ivory and bone buckles were being used.

Type 7

A D-shaped buckle of unusually narrow form, one copper-alloy example from the cemetery.

Type 8

Buckle with swivelling arm or ‘locking’ buckle. Locking buckle with rectangular frame, one copper-alloy example from the cloister walk/garth. A wooden statue of St Roch in the Rijksmuseum in Amsterdam attributed to the circle of Dries Holthuys who was active in Cleves c 1492–1508 clearly depicts a buckle of this type on a belt.^^[[8]](#footnote-8)^^ It is unclear how this distinctive type of buckle functioned, as they could not ‘lock’. One possibility is that the arm was used as a hook from which to suspend a purse.^^[[9]](#footnote-9)^^ Probably late 15th–16th century. Purse frames are known from several friary sites and the purses could have been used to hold collected alms.

## APPENDIX 3: CATALOGUE OF BUCKLES FROM 2016–17 EXCAVATIONS

*By* Craig Cessford, Andrew Hall, Ian Riddler and Justin Wiles

**F.216**: copper-alloy buckle with oval or D-shaped lipped frame with V-shaped notch for the pin and slightly offset opposing bar. The plate is formed from folded rectangular copper-alloy sheet, with a rectangular slot for the pin. The plate is recessed for the frame. There is an oval aperture with groove on the reverse and obverse at the end of the slightly tapering plate. This has two small rivets at the back end, but there is also an additional, later, centrally placed crude rivet probably to affect a repair. This appears to have a plate normally associated with frames with forked spacers. 47 x 30 mm, 13 g, typologically 14th century. Leather strap 20 mm wide, 3 mm thick.

**F.265**: copper-alloy buckle with oval, lipped frame. The pin and rectangular plate are intact. The frame has a V-shaped seat or notch for the pin on the outside edge with the opposing bar slightly offset to the back end of the frame. The plate is formed of a folded sheet with a rectangular slot for the pin and two rivets at the end of the plate. The pin is flanged with a transverse ridge. Little evidence of use wear. 38 x 24 mm, 9 g, typologically mid-14th–mid 15th century. Leather strap 15–18 mm wide, 3 mm thick.

**F.302**: two fragments of an annular copper-alloy buckle, with partial pin attached. Heavily distorted and fragmentary. Diameter approximately 35 mm, 7 g. Found with a copper-alloy rectangular mount of shallow pyramidal form, with a single integral rivet, attached to a double layer of leather strap. 11 x 12 mm, 2 g, typologically 14th–early 15th century. Leather strap 17 mm wide, 2–3 mm thick

**F.311**: iron buckle with circular frame and pin in poor condition. 41 mm diameter, 18 g, typologically medieval. Leather strap width unknown 3–4 mm thick.

**F.314**: skilfully produced near complete buckle with integral plate, made from elephant ivory with Schreger lines visible. Oval frame with a tongue groove and moulded lateral knops in front of the tongue rest. The integral buckle plate is rectangular in shape and is bifurcated along most of its length, allowing a strap to be secured with two rectangular headed copper-alloy rivets. The copper-alloy buckle pin is secured on a shaft, also of copper alloy, which runs laterally through most of the buckle from one side, but does not emerge on the other side. The upper surface of the buckle is lightly embellished with spaced triangular notches set between three sets of narrow parallel lines, providing a cable-like effect. This buckle and the example from F.347 can be dated by reference to the form of their frames and plates. Oval lipped frames occur in London contexts within ceramic phases 11–12, and predominantly in phase 11, of c 1350–1400.^^[[10]](#footnote-10)^^

Buckles made from elephant ivory form a much more exclusive commodity than those made of bone, with a specific provenance. Late-medieval buckles of this material are likely to have been made in France, and most probably in Paris, a centre of medieval elephant-ivory production. From the late 13th century onwards, Paris and other workshops in France were major producers of objects of elephant ivory, whilst comparatively little was produced in this material elsewhere in northern Europe, or in England.^^[[11]](#footnote-11)^^ In a French text of c 1260 that describes Parisian workshops for ivory, as well as for the working of bone, horn and other materials, the patenostriers are listed as producing buttons and buckles, as well as rosary beads.^^[[12]](#footnote-12)^^ Accordingly, it seems likely that the ivory buckle was made in a French workshop, probably in Paris, before eventually finding its way to Cambridge.

**F.331**: copper-alloy oval or D-shaped buckle, the frame thickens towards the middle and has a rectangular notch or recess for the pin. The opposing bar is slightly offset. The pin is intact and appears to have fused tightly within the frame notch. Similar buckles from London are dated to the 14th century.^^[[13]](#footnote-13)^^ This buckle and pin display very little use wear. 23 x 31 mm, 9 g. A crescent-shaped copper-alloy mount was found in association with the buckle, attached to the strap by two integral round flatheaded rivets. 19.2 x 12.3 mm, 2 g. The lower strap at the mount has a straight end with cropped corners indicating it to be a terminal. The upper strap, closest to the mount, also appears to have slightly tapering sides, the end is now slightly broken but had been straight, which would suggest a second terminal, rather than a join to extend the length of the strap. Width 16 mm, 3 mm thick, bovine. At the buckle pin the strap is 7 mm wide and 3 mm thick.

**F.332**: copper-alloy oval or D-shaped buckle. The frame thickens towards the middle and has a deep rectangular notch or recess for the pin. Slightly offset and narrowed opposing bar. The pin has a moulded, ridged section to the upper surface towards the base. Traces of leather present around the pin. Similar to F.331. 22 x 29 mm, 10 g, typologically 14th century. Leather strap 14 mm wide, 2–4 mm thick.

**F.333**: iron buckle in poor condition, heavily corroded. Possibly oval or D-shaped frame. 38 x 33 mm, 10 g, typologically medieval.

**F.334**: copper-alloy buckle with oval or D-shaped frame, with pin and rectangular plate intact. The simple frame has a V-shaped seat or notch for the pin on the outside edge with the opposing bar slightly offset. The plate is formed from a folded sheet with a rectangular slot for the pin. There are a pair of short V-shaped notches on the front from the pin slot towards the back end of the plate. The strap is attached with two round rivets positioned towards the end of the plate. The pin has a small flange. 43 x 19 mm, 10 g, typologically mid-14th–mid-15th century. Leather strap 15–20 mm wide, 3 mm thick.

**F.336**: copper-alloy buckle with oval or D-shaped frame, the pin and short rectangular plate are intact with leather adhering to the plate. The frame is lipped on the outside edge with a V-shaped notch for the pin. The opposing bar is slightly offset. The plate is formed from a rectangular sheet of copper alloy with a rectangular notch for the pin. The upper surface of the plate may have traces of tinning or another coating. The pin has a flanged transverse ridge. There are two rivet holes towards the back of the plate to secure the leather strap within. 31 x 21 mm, 8 g, typologically 14th century. Leather strap 12–14 mm wide, 4 mm thick. Textile present.

**F.343**: iron buckle frame in heavily corroded and fragmentary condition. D-shaped form, with possible pin attached. 35 x 46 mm, 21 g, medieval.

**F.344**: copper-alloy D-shaped buckle of unusually narrow form. The frame has a rectangular notch or recess for the pin. The pin has a slight transverse ridge. An unusual buckle possibly not used with a girdle, although its position within the grave contradicts this. A similar buckle made of iron is recorded from York.^^[[14]](#footnote-14)^^ 30 x 20 mm, 5 g, typologically mid-14th–15th century. Leather strap width unknown, 3–4 mm thick. Textile present.

**F.347**: buckle made of animal bone, to a similar but slightly different design than the elephant-ivory example from F.314. Oval frame including a tongue rest, but lacks any lateral knops. The thin copper-alloy pin was secured on a lateral shaft of iron, which no longer survives. This was inserted laterally through one side of the buckle, as with the ivory example. The integral plate has lightly curved edges and the upper surface is decorated with a lateral groove and two raised mouldings, with bands of triple incised lateral lines set just below the pin and along the inner edge of the plate. The plate is bifurcated and was secured to a strap with three small copper-alloy rivets.

Late-medieval bone buckles are known from France in particular.^^[[15]](#footnote-15)^^ They occur in several forms, both with and without integral plates. Of the latter type, those with long rectangular plates are common.^^[[16]](#footnote-16)^^ Buckles with shorter integral plates, closer in form to the pair from Cambridge, have been found at Goltho, Douai (France) and York, and there are also several unprovenanced examples.^^[[17]](#footnote-17)^^ One of the Goltho buckles is unstratified, whilst the other, which forms a close parallel for the Cambridge bone buckle, was found on cobbles lying beside a building abandoned in the late 14th or early 15th century.^^[[18]](#footnote-18)^^ The Douai bone buckle now lacks its frame and its iron spindle passes through both edges of the plate, but its simple decoration is similar to the Cambridge bone buckle; it came from a context of the second half of the 14th century.^^[[19]](#footnote-19)^^ The York buckle has no precise provenance.^^[[20]](#footnote-20)^^ The distribution of these bone buckles, with finds from Goltho and York, suggests that they may well have been made in England, quite possibly in northern England. An example from Wharram Percy (North Yorkshire) is probably of an earlier 10th–12th century type, despite being found in a late-medieval context.^^[[21]](#footnote-21)^^

**F.348**: heavily corroded iron buckle with plate and pin. Oval or D-shaped frame with rectangular plate. Leather strap attached by two rivets. 41 x 29 mm, 16 g. 14th–15th century. Leather strap 28 mm wide, 4 mm thick. Textile present, probably relatively coarse animal fibre.

**F.352**: copper-alloy buckle with oval or D-shaped frame. The pin and rectangular plate are intact. The frame is lipped on the outside edge with a V-shaped seat or notch for the pin. It appears slightly misshapen. The opposing bar is offset. The plate is formed from folded rectangular sheet copper alloy with rectangular notch for pin. The buckle plate is also recessed for the frame. The end of the plate has a centrally positioned aperture of circular form with angled grove pointing towards the pin on both the upper and lower surfaces. The back end of the plate is also slightly concave in shape with two protruding rivets fixing a leather strap fragment within the plate. No visible decoration to the upper surface of the plate. The pin has distinct flanges. The plate matches a type used on buckles with composited rigid plates, however, this buckle frame lacks the integral forked spacer that normally accompanies this type of buckle.^^[[22]](#footnote-22)^^ 47 x 22 mm, 12 g, this appears to be an interesting hybrid typologically dating to the later 14th century. Leather strap 16 mm wide, 3–4 mm thick.

**F.367**: copper-alloy buckle with rectangular frame and rectangular sheet copper-alloy plate intact. The frame has a thick outside edge with two filed grooves towards the top and bottom and three grooves towards the middle, the central one acting as a seat or guide for the pin. The sides of the frame are very thin and slightly convex in form. The pin is intact and of simple form. The plate is decorated to the upper surface with three parallel grooves towards the back edge and there is the suggestion of parallel diagonal lines to the reverse. The plate is attached to a leather strap with two rivets towards the rear of the plate. Similar to examples for both York and London.^^[[23]](#footnote-23)^^ Little use wear. Frame 20 x 21 mm, plate 25 x 18 mm, 14 g, typologically 14th century. Leather strap 17–19 mm wide, 3–5 mm thick, bovine.

**F.191**: cast copper-alloy double oval frame buckle, with the frame thickening to middle on either side. The central bar is slightly narrower and projects slightly above and below the frame. There are filing marks present along the outside edge of the frame. There appears not to be a seat or notch for the pin. The pin is missing; however the trapezoidal sheet plate is present, there are two rivets towards the back end with leather still adhering within the plate. 45 x 40 mm, 16 g. Late 14th–15th century. Leather strap 30 mm wide, 4 mm thick

**F.230**: small double oval copper-alloy buckle frame with decorative grooves on both sides of the frame and intact pin. 28 x 24 mm, 7 g, typologically 14th–early 15th century. Leather strap 16 mm wide, 3 mm thick

**F.260**: disturbed, length of heavily corroded copper-alloy pin, 1 g. Leather strap present.

**F.310**: small double oval copper-alloy buckle frame with slightly asymmetrical loops. The upper portion of one of the loops shows signs of wear. Traces what appears to be an iron pin remain. Similar buckles from London have been suggested to be shoe buckles,^^[[24]](#footnote-24)^^ however, the position within the grave indicates it was a girdle buckle. 25 x 19 mm, 3 g, typologically 14th–early 15th century. Leather strap 9 mm wide, 2 mm thick.

## APPENDIX 4: CATALOGUE OF BUCKLES FROM THE 1908–09 INVESTIGATIONS

*By* Craig Cessford and Andrew Hall

In 1908–9 during building work at the site of the friary, some copper-alloy buckles were found associated with skeletons. These are mentioned in a publication on the human remains. In 1908, ‘Two very plain copper or brass buckles were found. To these a few crumbling relics of what might have been leather girdles were still adherent’, and in 1909, ‘several buckles and remnants of clothing’ were recovered.^^[[25]](#footnote-25)^^ In 1910, ‘Six bronze buckles with fragments of leather belts attached’ found ‘on the site of the Austin friary, Benet Street, Cambridge,’ were donated to the Museum of General and Local Archaeology and of Ethnology of the University of Cambridge, now the Museum of Archaeology and Anthropology, by the Cambridge Antiquarian Society.^^[[26]](#footnote-26)^^

Later, in 1923, the museum catalogue records that ‘3 bronze buckles & 1 strap end … all found with skeletons at Friars (Peas Hill)’ were acquired from the Redfern collection. These were part of ‘an important series of local antiquities, of the Bronze Age and later, which he [Redfern] had collected’ presented to the museum by Lady Ryan of Hintlesham Hall, in his memory.^^[[27]](#footnote-27)^^

As buckles from the friary came into the possession of both the Cambridge Antiquarian Society and a local antiquarian, it is possible that more buckles were discovered in 1908–09 that did not end up in the Museum of Archaeology and Anthropology. Some of the skeletons discovered in 1908–9 are located on a plan drawn up at the time by Edward A Schneider, the Clerk of Works Architectural for the University of Cambridge.^^[[28]](#footnote-28)^^ Based on the evidence from the more recent investigations, these represent burials in the cloister walk and garth and the skulls still survive in the Duckworth Collection of the University of Cambridge. Unfortunately, it is not possible to connect particular buckles to individual skeletons, or the physical remains to the skeletons depicted on the plan.

**1923.1597D**: strap-end of simple rectangular form, with a slight taper towards the terminal. The open end has an aperture with an angled groove. Two rivets hold a length of leather strap within. 28 x 24 mm, 9.5 g, typologically medieval.

**41923.1597B**: buckle of D-shaped frame, with rectangular sheet plate. The frame is thin with offset bar and rectangular notch or seat for the pin, which is present. The plate is undecorated, recessed for the frame with a notch for the pin. A pair of rivets hold a surviving length of leather strap within the plate. 43 x 29 mm, 15.7 g, typologically 14th–15th century.

**1910.273**: small, simple buckle with a D-shaped frame. Two slight ridges on the outside edge of the frame near to the bar, and a notch for the pin, which is broken off half way. The sheet plate is rectangular, slightly recessed for the frame, with two rivets. 38 x 21 mm, 8.25 g, typologically 14th–15th century.

**1923.1597C**: buckle with a D-shaped, slightly angular frame, triangular notch/seat for pin. The rectangular sheet plate is slightly recessed for the frame, with rectangular slot for the pin and two rivets intact. 48 x 24 mm, 13 g, typologically 14th–15th century.

**1910.274**: small locking buckle, very similar to 1928.964. The locking arm is broken. A small fragment of leather strap of 11mm width is attached. 30 x 17 mm, 10 g, typologically later medieval.

**1910.271**: oval or D-shaped buckle frame with an off-set bar. The frame thickens towards the pin notch/seat. The pin is intact and has two transverse ridges, forming a grip towards its base. 32 x 27 mm, 12.6 g. Several fragments of leather strap of 22 mm width were found in association with the buckle, including one attached to the pin. A further fragment has a crescent-shaped mount: 23 x 13 mm, 3.4 g, attached vertically across the strap with two rivets. The same fragment has several additional pierced holes, suggestive of further absent mounts, which might have been overlooked in 1980–9 or may have become separated subsequently. 32 x 27 mm, 12.6 g. The mount(s) act as a fixing device, replacing the buckle plate, with the mount’s rivets fixing two layers of girdle strap forming a loop around the buckle bar. Typologically medieval.

**1910.275B?**: robust buckle with integral rectangular loop for strap. The frame thickens towards the central pin notch/seat. The pin has chamfered edges to the upper surface and a single transverse ridge. 34 x 32 mm, 21 g. Two fragments of leather strap of 24 mm width also present. Found with a small mount in the form of two vertically stacked crescents, each with a central rivet: 22 x 10mm, 1.8g. As with 1910.271 the crescent-shaped mount obviates the need for a buckle plate. Typologically medieval.

**1910.270**: finely made large annular buckle/brooch with a circular frame with chamfered sides. There is a decorative protrusion from the frame with a moulded collar and knop, at the point at which pin notch/seat is situated. On the opposing side is a narrow constriction for the pin. This is probably a brooch, but fragments of leather around the pin suggest that in its ultimate role it was repurposed as a buckler. The pin is formed from rolled sheet and is almost certainly a replacement. A less decorative parallel is recorded from London.^^[[29]](#footnote-29)^^ 58 x 45 mm, 19.5 g. Typologically 14th–15th century.

## APPENDIX 5: THE LEATHER GIRDLES AND TEXTILES

*By* Esther Cameron (textiles) and Quita Mould (leather)

Although it is principally the buckles that survive at the Cambridge friary, these act as a proxy for clothed burial and the textual evidence makes it clear that the black leather girdle and cowl were the important elements of Augustinian costume. Although less well-preserved leather and textiles did survive. Seventeen of the buckles from the 2016–17 excavations, plus five recovered in 1908–9, had leather associated with them. Where sufficient leather survived for the form to be discernible all came from parallel-sided straps, which may be assumed to be girdles worn around the waist or hips and are probably all vegetable tanned cattle leather. The straps found in 2016–17 appear to be undecorated and were 7–30 mm wide, with most 15–20 mm wide. They are 2–5 mm thick, with the majority 3–4 mm thick. The straps from the 1908–9 excavations are generally 20–21 mm wide. As friars’ girdles should be one and a half to two fingers wide and a finger is 3⁄4 of an inch this is c 28–38 mm. This indicates that the Cambridge girdles were narrower than stipulated.

Three textile fragments associated with buckles from the recent excavations were poorly preserved. A better-preserved textile fragment from the 1908–9 excavations is a plain tabby weave, most probably woollen. Its surface is slightly burred, which might be due to wear or possibly a result of ‘fulling’. It is a balanced weave, both systems with approximately 11 threads per cm, z-spun threads. This is different to the more recently discovered textiles, one has s-spun threads while the other has s-spun in one direction, z-spun in the other, possibly a twill rather than a tabby weave, although the density of weave (possibly ten in one direction, eight in the other) is quite close to the 1908–9 fragment thread count.

## BIBLIOGRAPHY

Andrews, D D and Milne, G 1979, *Wharram: A Study of Settlement on the Yorkshire Wolds. Volume* **I***. Domestic Settlement, 1: Areas 10 and 6*, Soc Mediev Archaeol Monogr **8**.

Anonymous, 1914, ‘Appendix I. 1 List of Accessions from J January 1 to December 31 1910’, *Twenty-Eighth Annual Report of the Antiquarian Committee to the Senate and Lists of Accessions for the Years 1910, 1911, 1912*, Cambridge: University of Cambridge, 9–15. Available online at: <*https://maa.cam.ac.uk/maa/wp-content/uploads/2016/06/AR1912-28th.pdf*> [accessed 15th April 2021].

Anonymous, 1924, ‘Appendix I: List of Accessions from 1 January to 31 December 1923’, in *Report of the Board of Archaeological and Anthropological Studies 14 May 1924* (unpaginated), Cambridge: University of Cambridge,. Available online at: <*https://maa.cam.ac.uk/maa/wp-content/uploads/2016/06/AR1923-39th.pdf*> [accessed 15th April 2021].

Beresford, G 1975, *The Medieval Clay-Land Village: Excavations at Goltho and Barton Blount*, Soc for Mediev Archaeol Monogr **6**.

Cassels, A K 2013, The Social Significance of Late Medieval Dress Accessories, (unpubl PhD thesis, University of Sheffield).

Chaoui-Derieux, D 2010, ‘Socio-economic and cultural implications in medieval society: the unpublished collections of the Region of Douai (France)’, in A Legrand-Pineau, I Sidéra, N Buc et al (eds), *Ancient and Modern Bone Artefacts from America to Russia: Cultural, Technological and Functional Signatures*, Brit Archaeol Rep, Inter Ser **2136**, 65–70.

Chazottes, M-A and Thuaudet, O 2014, ‘L’utilisation des matières dures d’origine animale dans la production d’accessoires de la ceinture à la fin du Moyen Âge: quelques exemples provençaux’, *Archéologie du Midi Médiéval* **32**, 183–98.

Duckworth, W L H and Pocock, W I 1910, ‘On the human bones found on the site of the Augustinian Friary, Bene’t Street, Cambridge’, *Proc Cambridge Antiq Soc* **14**, 7–38.

Egan, G and Pritchard, F 1991, *Dress Accessories c. 1150–c. 1450. Medieval Finds from Excavations in London* **3**, London: Her Majesty’s Stationery Office.

Gaborit-Chopin, D 2003, *Ivoires Médiévaux Ve–XVe Siècle, Musée du Louvre, Département des Objets d’Art, Paris*, Paris: Éditions de la Réunion des Musées Nationaux.

MacGregor, A 1985, *Bone, Antler, Ivory and Horn. The Technology of Skeletal Materials since the Roman Period*, London: Croom Helm.

MacGregor, A 1991, ‘Antler, bone and horn’, in J Blair and N Ramsay (eds), *English Medieval Industries*, London: Hambledon Press, 355–78.

Ottaway, P and Rogers, N 2002, *Craft, Industry and Everyday Life: Finds from Medieval York*. (Archaeology of York **17/15**.) York: Council for British Archaeology.

Riddler, I D 2012, ‘The late Saxon material culture’, in S Wrathmell (ed), *A History of Wharram Percy and its Neighbours*, Wharram. A Study of Settlement on the Yorkshire Wolds **XIII**, York University Archaeological Publications **15**, 196–203.

Standley, E R 2010, Trinkets and Charms: The Use, Meaning and Significance of Later Medieval and Early Post-medieval Dress Accessories, (unpubl PhD thesis, Durham University).

Stratford, N 1987, ‘Gothic ivory carving in England’, in J Alexander and P Binski (eds), *Age of Chivalry. Art in Plantagenet England 1200–1400*, London: Royal Academy of Arts, 107–13.

Waterman, D M 1959, ‘Late Saxon, Viking and early medieval finds from York’, *Archaeologia* **97**, 59–105.

Williams, D 2018, *Copper-alloy Purse Components: a New Classification using Finds from England and Wales Recorded by the Portable Antiquities Schem*e, London: Finds Research Group.

1. Egan and Pritchard 1991, 78–82. More D-shaped examples include nos 324, 326, 329, 330. [↑](#footnote-ref-1)
2. Ibid, 78–82. [↑](#footnote-ref-2)
3. Ibid, 82–3. [↑](#footnote-ref-3)
4. Ibid, 70, 102–03. [↑](#footnote-ref-4)
5. Standley 2010, 72. [↑](#footnote-ref-5)
6. Cassells 2013, 85–86, 107–08. [↑](#footnote-ref-6)
7. Egan and Pritchard 1991, 76–7. [↑](#footnote-ref-7)
8. <*https://www.rijksmuseum.nl/en/collection/BK-NM-24.>* [Accessed 20-06-2019] [↑](#footnote-ref-8)
9. Williams 2018. [↑](#footnote-ref-9)
10. Egan and Pritchard 1991, 70, 74–5. [↑](#footnote-ref-10)
11. Stratford 1987, 108–9; Gaborit-Chopin 2003, 21–2. [↑](#footnote-ref-11)
12. MacGregor 1991, 377; Gaborit-Chopin 2003, 266–69. [↑](#footnote-ref-12)
13. Egan and Pritchard 1991, 70. [↑](#footnote-ref-13)
14. Ottaway and Rodgers 2002, 2892 [↑](#footnote-ref-14)
15. Chazottes and Thuaudet 2014. [↑](#footnote-ref-15)
16. Ibid, 189–90, fig 3. [↑](#footnote-ref-16)
17. Ibid, fig 5. [↑](#footnote-ref-17)
18. Beresford 1975, 26, 77. [↑](#footnote-ref-18)
19. Chaoui-Derieux 2010, 67, fig 9. [↑](#footnote-ref-19)
20. Waterman 1959, fig 19.7. [↑](#footnote-ref-20)
21. Andrews and Milne 1979, 128 and fig 70.31; MacGregor 1985, 105; Riddler 2012, 197 [↑](#footnote-ref-21)
22. Egan and Pritchard 1991, 79. [↑](#footnote-ref-22)
23. Egan and Pritchard 1991, 96; Ottaway and Rogers 2002, 2839. [↑](#footnote-ref-23)
24. Ibid, 86. [↑](#footnote-ref-24)
25. Duckworth and Pocock 1910, 24. [↑](#footnote-ref-25)
26. Anon 1914, 10. [↑](#footnote-ref-26)
27. Anon 1924. William Beales Redfern/Redfarn (1840–1923) was born in Cambridge in 1840, and from the 1870s onwards was a keen antiquarian publishing several articles and books. Redfern had a private museum with a large collection of material. Lady Ryan was Ellen Amelia Ryan nee Ellis (1860–1935), who had been born in Cambridge. [↑](#footnote-ref-27)
28. Duckworth and Pocock 1910, facing p 38. [↑](#footnote-ref-28)
29. Egan and Pritchard 1991, 61 no 212. [↑](#footnote-ref-29)
